# Degraders in epigenetic therapy: PROTACs and beyond

**DOI:** 10.7150/thno.92526

**Published:** 2024-01-27

**Authors:** Xing-Jie Dai, Shi-Kun Ji, Meng-Jie Fu, Gao-Zhi Liu, Hui-Min Liu, Shao-Peng Wang, Liang Shen, Ning Wang, Piet Herdewijn, Yi-Chao Zheng, Sai-Qi Wang, Xiao-Bing Chen

**Affiliations:** 1 Department of Oncology, the Affiliated Cancer Hospital of Zhengzhou University & Henan Cancer Hospital, Zhengzhou University, Zhengzhou, China.; 2 Department of Oncology, the Affiliated Cancer Hospital of Zhengzhou University & Henan Cancer Hospital, Henan Engineering Research Center of Precision Therapy of Gastrointestinal Cancer & Zhengzhou Key Laboratory for Precision Therapy of Gastrointestinal Cancer, Zhengzhou, China.; 3 Key Laboratory of Advanced Drug Preparation Technologies, Ministry of Education, China; State Key Laboratory of Esophageal Cancer Prevention & Treatment; Key Laboratory of Henan Province for Drug Quality and Evaluation; Institute of Drug Discovery and Development; School of Pharmaceutical Sciences, Zhengzhou University, Zhengzhou, China.; 4 XNA platform, School of Pharmaceutical Sciences, Zhengzhou University, Zhengzhou, China.; 5 The School of Chinese Medicine, The University of Hong Kong, Pokfulam, Hong Kong, China.; 6 Rega Institute for Medical Research, Medicinal Chemistry, KU Leuven, Herestraat 49-Box 1041, 3000 Leuven, Belgium.

**Keywords:** PROTACs, epigenetics, anti-cancer, drug design, protein degrader

## Abstract

Epigenetics refers to the reversible process through which changes in gene expression occur without changing the nucleotide sequence of DNA. The process is currently gaining prominence as a pivotal objective in the treatment of cancers and other ailments. Numerous drugs that target epigenetic mechanisms have obtained approval from the Food and Drug Administration (FDA) for the therapeutic intervention of diverse diseases; many have drawbacks, such as limited applicability, toxicity, and resistance. Since the discovery of the first proteolysis-targeting chimeras (PROTACs) in 2001, studies on targeted protein degradation (TPD)—encompassing PROTACs, molecular glue (MG), hydrophobic tagging (HyT), degradation TAG (dTAG), Trim-Away, a specific and non-genetic inhibitor of apoptosis protein (IAP)-dependent protein eraser (SNIPER), antibody-PROTACs (Ab-PROTACs), and other lysosome-based strategies—have achieved remarkable progress. In this review, we comprehensively highlight the small-molecule degraders beyond PROTACs that could achieve the degradation of epigenetic proteins (including bromodomain-containing protein-related targets, histone acetylation/deacetylation-related targets, histone methylation/demethylation related targets, and other epigenetic targets) via proteasomal or lysosomal pathways. The present difficulties and forthcoming prospects in this domain are also deliberated upon, which may be valuable for medicinal chemists when developing more potent, selective, and drug-like epigenetic drugs for clinical applications.

## 1. Introduction

Epigenetics refers to the reversible process through which changes in gene expression occur without changing the nucleotide sequence of DNA. These can encompass DNA/RNA modifications, histone modifications, chromatin remodeling, post-translational modifications, and non-coding RNA interference 1. Epigenetics contributes significantly to cell growth, development, and differentiation through dynamic regulation of gene transcription and genomic stability, which is carried out by “writers” (enzymes that deposit modifications, including DNA methyltransferases (DNMTs), histone methyltransferases (HMTs), histone acetyltransferases (HATs) and ubiquitin E3 ligases), “readers” (proteins that recognize and bind epigenetic modifications, including bromodomains(BRDs), chromodomain proteins, and methyl-CpG binding proteins), and erasers (enzymes that remove modifications, including DNA demethylases, histone deacetylases (HDACs), histone demethylases (KDMs) and deubiquitinating enzymes) 2. Dysregulation of these epigenetic modifications is associated with the onset and progression of various diseases, such as cancer and autoimmune, cardiovascular, and neurological disorders 3. Over the course of recent decades, significant advancements have been achieved in discovering novel epigenetic drug targets, uncovering the role of epigenetic mechanisms in various complex disorders, and developing tools and clinical epigenetic modulators 4. The most commonly investigated and FDA-approved epigenetic drugs are DNA methyltransferase inhibitors (DNMTis) and histone deacetylase inhibitors (HDACis) 5. Collectively, epigenetic targets provide significant new routes for successful drug discovery research.

**Figure 1:**
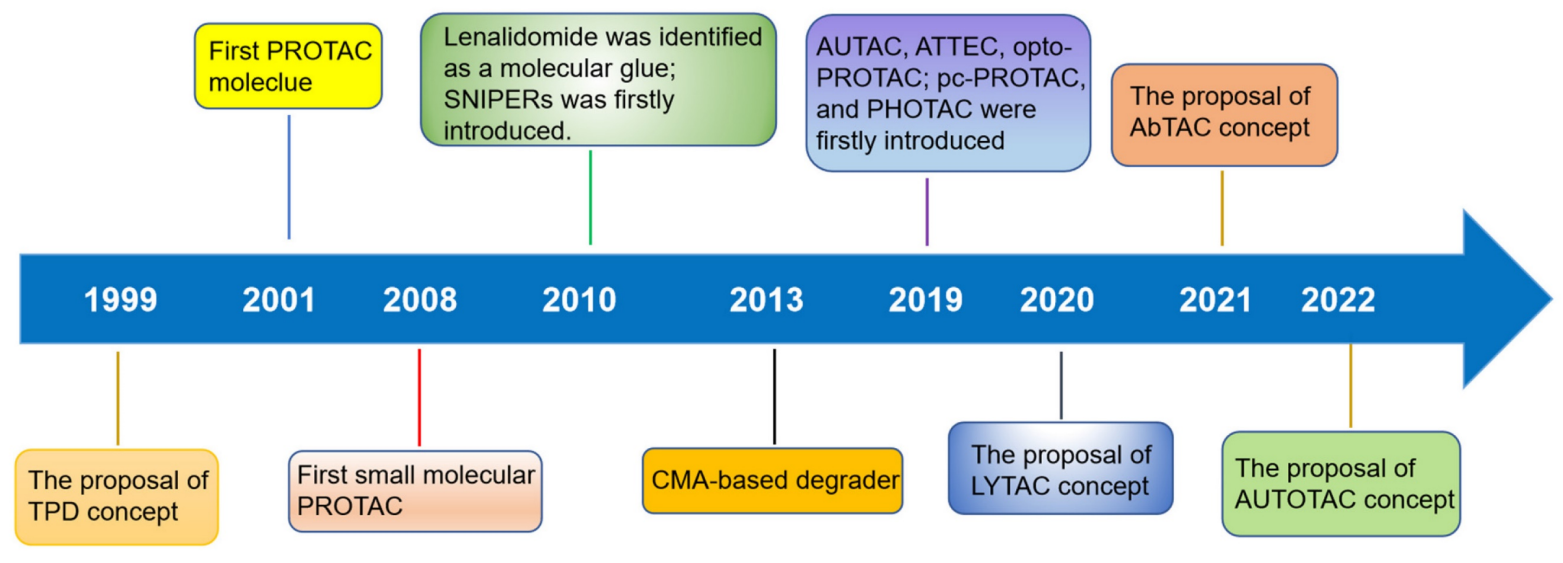
Timeline and milestones for the development of degrader technologies.

TPD, via the proteasomal and lysosomal pathways, has represented a promising new research direction in the realm of pharmaceutical research and development over the last two decades as an important complement and alternative to traditional inhibitor-based therapeutics (Figure 1) 6. PROTACs were first introduced in 2001 by Crew *et al*., represent degraders that hijack the endogenous ubiquitin-proteasome system (UPS) 7. Herein, we provide a concise description of the UPS pathway and how it relates to PROTACs.

**Figure 2:**
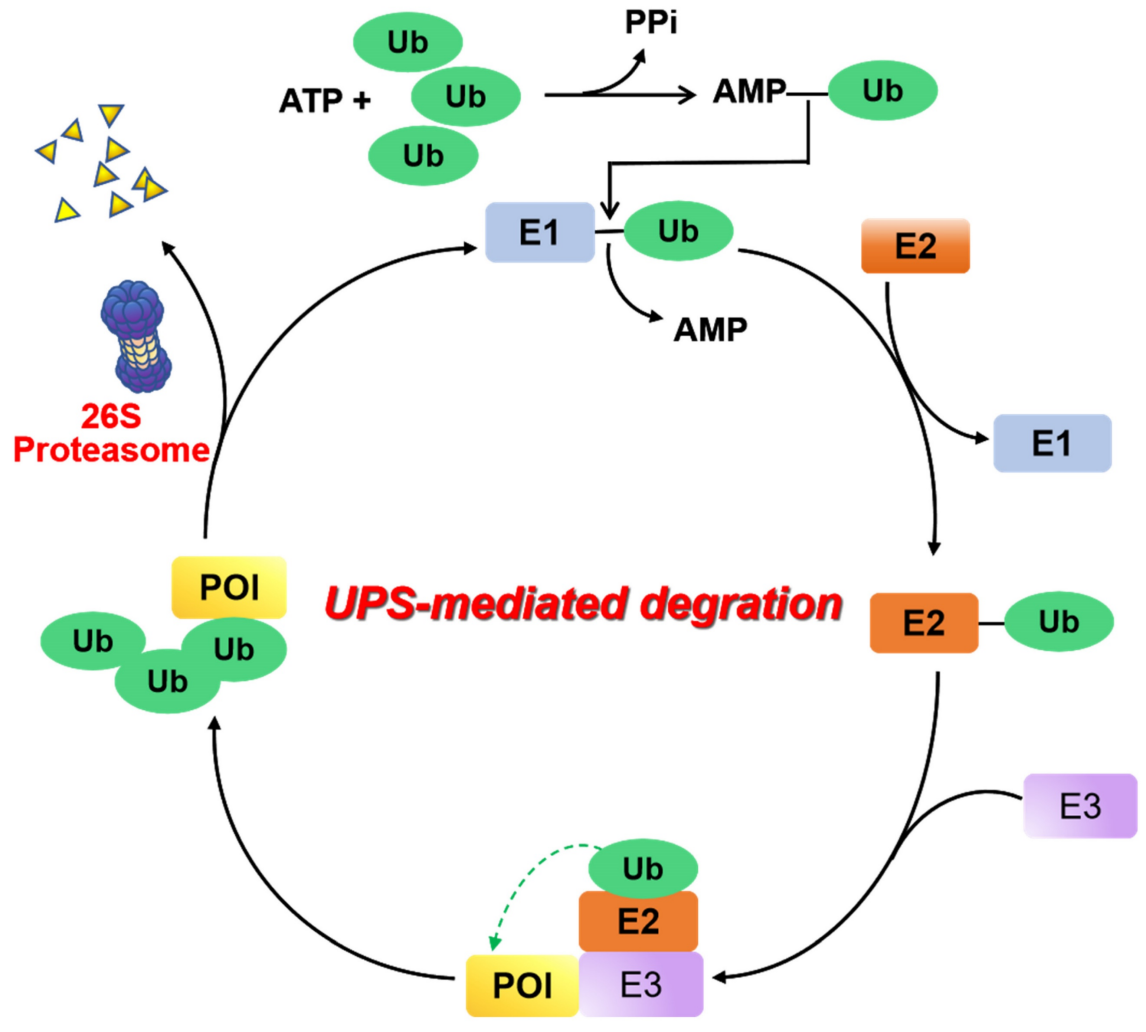
Mechanism of UPS. E1 ubiquitin activating enzyme catalyzes the first step of activation of ubiquitin protein (Ub) in an ATP-dependent manner. Following this, Ub is transferred to an E2 ubiquitin binding enzyme, and subsequently relies on an E3 ubiquitin ligase to deliver Ub to the protein of interest (POI). Ultimately, the poly-ubiquitinated proteins are recognized and degraded by the 26S proteasome.

UPS is a major proteolysis system that controls intracellular protein degradation as a part of normal cellular maintenance processes 8. This pathway is mediated by a three-step enzymatic cascade: a ubiquitin-activating enzyme (E1), a ubiquitin-conjugating enzyme (E2), and a ubiquitin-protein ligase (E3) 9. First, the process of covalently bonding the carboxyl-terminus of a Ub polypeptide consisting of 76 amino acids to a cysteine residue found on ubiquitin-E1 is facilitated by an ATP-dependent mechanism. The transfer of the ubiquitin molecule from the E1 enzyme to the catalytic cysteine residue of the E2 enzyme is accomplished by *trans*-thioesterification. Next, an E3 ligase binds the corresponding ubiquitin-E2 conjugate and transfers ubiquitin to the lysine residue of the substrate protein. When the substrate protein is modified with multiple ubiquitins (“poly-ubiquitination”), the 26S proteasome can be identified and undergo degradation of this substrate protein, and the ubiquitin proteins are then released and recycled (Figure 2).

**Figure 3:**
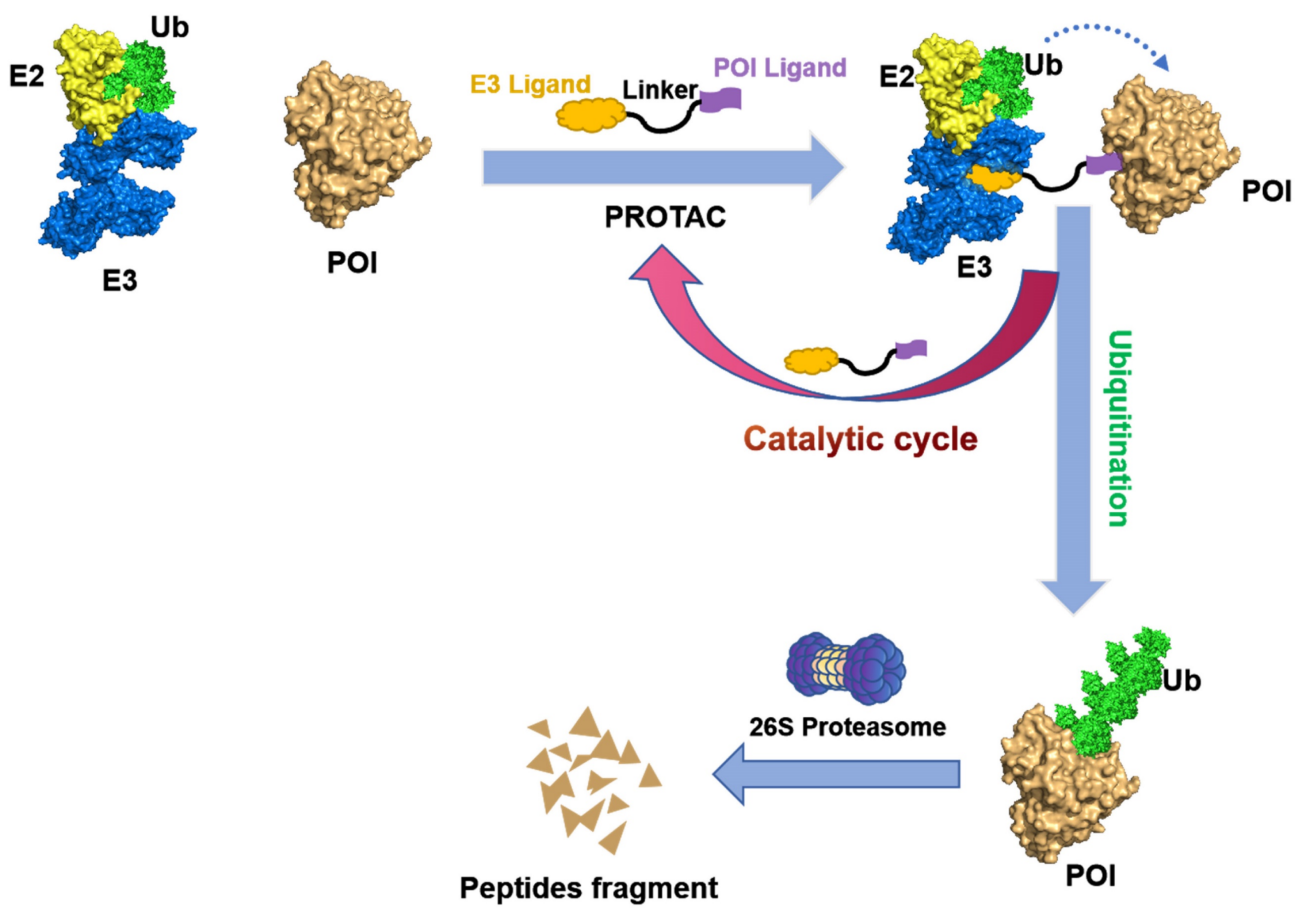
Mechanism of degradation by PROTAC. A PROTAC is composed of a warhead that specially targets the POI, a ligand that recruits the E3 ubiquitin ligase, and a linker that connects these components. Upon the PROTAC forms a ternary complex with the POI and E3 ligase, the E3 ligase facilitates the transfer of ubiquitins to the POI using an E2 ubiquitin-conjugating enzyme. Subsequently, the polyubiquitinated POI is recognized by the 26S proteasome and undergoes degradation. The PROTAC will be released during this process and can participate in subsequent rounds of degradation.

PROTACs are hetero-bifunctional small molecules that exploit the UPS machinery to induce the degradation of target proteins by redirecting the third step of the cascade. A PROTAC molecular consist of an E3 ubiquitin ligase-recruiting ligand, a ligand specific to the POI, and a suitable linker that connects the two components 7, 10, 11. As illustrated in Figure 3, PROTACs initiate a degradation cascade by establishing a durable ternary complex involving E3-PROTACs-POI; thus, the initiation of the poly-ubiquitination process followed by the subsequent degradation of the POI via the 26S proteasome. The PROTACs can then be liberated from the ternary complex and be recycled to enhance the ubiquitination and degradation of other POIs. Owing to this unique event-driven mechanism of action (MOA), PROTACs have several distinct advantages over small-molecule inhibitors (SMIs) that operate based on occupancy-driven MOAs. These include (1) low dosing, (2) improved target selectivity, (3) the capacity for overcoming drug resistance, (4) removal of the target proteins' enzymatic and non-enzymatic function, and (5) the modulation of non-druggable targets 12.

Traditional PROTACs typically exhibit unfavorable pharmacokinetics (PK) and lack tumor specificity, which may result in systemic toxicity due to their nonspecific distribution in normal tissues. Scientists are presently investigating approaches to enhance degradation activity in a cell-specific manner with the aim of minimizing undesirable side effects. To achieve tumor-targeting delivery and enhance the anticancer efficacy of PROTACs, several new PROTAC technologies including antibody-PROTAC conjugate (Ab-PROTAC), folate-PROTAC, aptamer-PROTAC conjugates, and poly-PROTAC nanoparticles, have been developed 13. Furthermore, the utilization of photo-PROTACs, which are coupled with a photolabile group to induce protein degradation through light stimulation, presents an alternative approach for achieving targeted drug effects within specific tissues or cells, thereby mitigating undesired side effects 14. The advancement in developing photo-PROTACs, such as photocaged PROTACs (pc-PROTACs) and photo-switchable PROTACs (photo-PROTACs), has unveiled novel opportunities for precise targeting of the disease-causing proteins. In addition, many other mechanisms of action associated with the tumor microenvironment have been utilized in the developed of innovative prodrug-based PROTACs (pro-PROTACs), including nitroreductase (NTR)-responsive PROTACs, radiotherapy-triggered PROTACs (RT-PROTACs), and ROS-responsive PROTACs. Furthermore, in-cell click-formed proteolysis targeting chimeras (CLIPTAC) was also proposed, which separates the bifunctional molecule into a tetrazine tagged thalidomide derivative and a *trans*-cyclooctene (TCO)-tagged POI ligand 15. These two components assemble into the PROTAC molecule via a rapid reaction within the cell. Owing to the reduced molecular weight and improved cell permeability of both components, showing significantly improved activity, selectivity, and drug-like properties than classical PROTACs. The forthcoming advancements in PROTAC technologies will prioritize attaining heightened selectivity, improved PK properties, augmented therapeutic efficacy, and diminished toxicity.

After 20 years of development, a variety of other strategies for TPD have also emerged, including MG, HyT, dTAG Trim-Away, SNIPER, autophagosome-tethering compound (ATTECs), autophagy-targeting chimeras (AUTACs), and others (Figure 1) 16. Many excellent reviews have been published regarding these novel targeted protein degradation technologies, so they will not be discussed here. Recently, the protein degradation technology has garnered interest from both academic and industrial sectors. Notably, a total of 16 degraders have progressed to clinical stages. Two examples of targeted therapies currently being investigated are ARV-110 (NCT03888612), which targets the androgen receptor (AR), and ARV-471 (NCT04072952), which targets the estrogen receptor (ER) 17 Thus far, various proteins linked with diseases have been effectively degraded —including nuclear receptors—AR, ER, et. al., epigenetic proteins—bromodomain-containing protein 4 (BRD4), epidermal growth factor receptor (EGFR), SWItch/sucrose non-fermentable (SWI/SNF) related, matrix associated, actin-dependent regulator of chromatin, subfamily A (SMARCA) et. al., protein kinases—Bruton's tyrosine kinase (BTK), anaplastic lymphoma kinase (ALK), mitogen-activated protein kinase (MEK), *et al*., other enzymes/proteins, and RNA 16.

Although there have been several excellent reviews on epigenetic degraders, most of them primarily focused on PROTAC-type degraders 18-22. Other protein degradation technologies beyond PROTACs, utilized for the degradation of epigenetic proteins, have also emerged as a current research hotspot. These alternatives hold the potential to overcome existing challenges associated with PROTACs, such as large molecular weight and PK issues. In the current review, we discuss a comprehensive state-of-the-art overview of therapeutic protein degraders that target epigenetic proteins involved in cancers and other diseases, focusing on the chemical structures, cellular and *in vivo* activities, and pharmacodynamics of PROTACs, as well as other novel types of protein degraders (Table 1). We also provide insights into the current limitations and future challenges of this field.

## 2. Bromodomain-containing proteins and related targets

### 2.1 Bromodomain and extra terminal (BET) proteins

The BET protein family encompasses four distinct proteins, namely bromodomain-containing protein 2 (BRD2), bromodomain-containing protein 3 (BRD3), BRD4, and the testis-specific bromodomain motif-containing protein (BRDT), which serve as epigenetic readers and fulfil the role of master transcription factors 102. Each BET protein is comprised of two conserved *N*-terminal bromodomains, namely BD1 and BD2, which are responsible for recognizing *N*-acetylated lysine residues present on histone tails, thereby exerting control over gene transcription 103. Notably, over the past ten years, several inhibitors designed to target BET proteins have been documented and subjected to clinical trials 104. Additionally, numerous small molecule-based PROTACs designed to specifically target BET proteins, have been developed in recent years 105.

#### 2.1.2 BRD4

**Figure 4:**
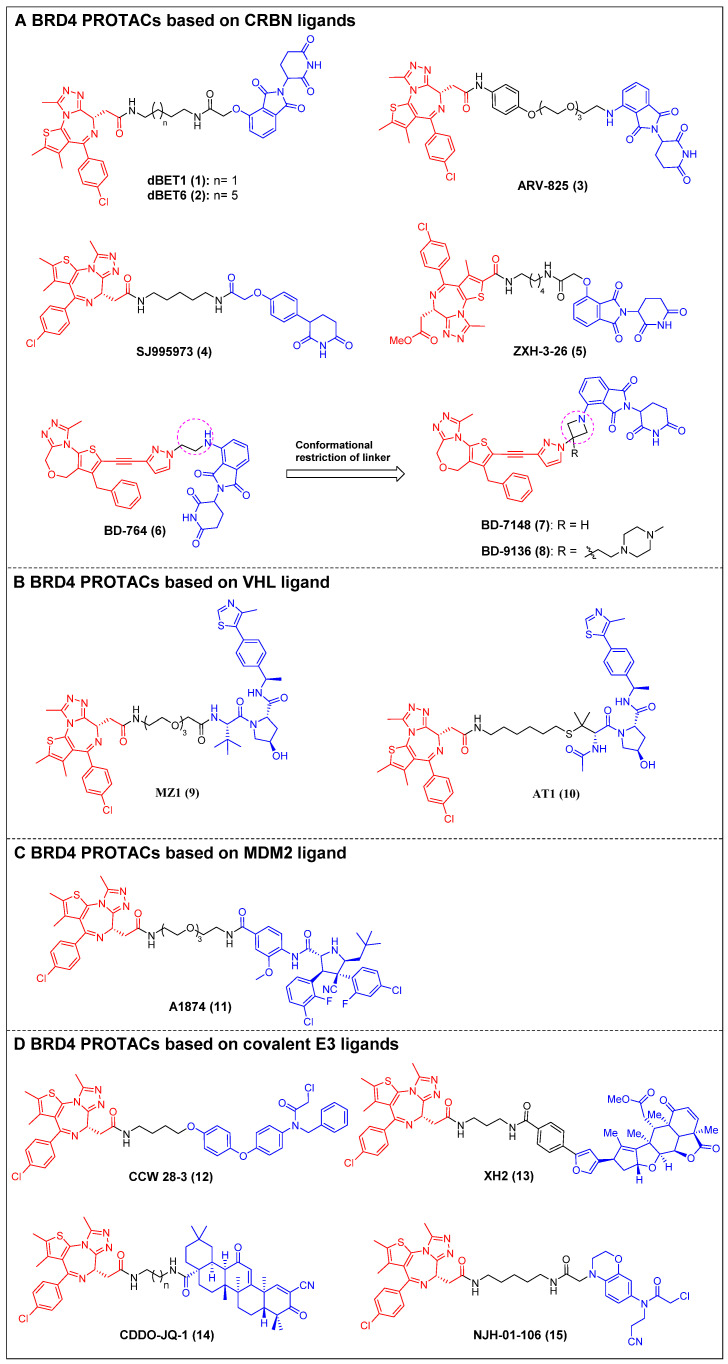
BRD4 PROTACs derived from JQ-1 (red for E3 ligand, blue for POI ligand and black for linker). BRD4 PROTACs based on cereblon (CRBN) ligands (A), von Hippel-Lindau (VHL) ligand (B), Mouse double minute 2 (MDM2) ligand (C), covalent E3 ligands (D).

The first reported BET-targeting PROTAC, dBET1 (compound 1) (Figure 4A), was established by Bradner *et al*. by conjugating the BRD inhibitor JQ-1 to the CRBN ligand through a flexible *N*-butyl-2-hydroxyacetamide linker 23. It specifically induced BRD4 degradation with a DC_50_ of 0.43 µM in SUM149 cells, and it delayed the progression of leukemia in mouse models of the disease. The significant reduction of the oncoproteins responsible for the pro-viral integration site of the Moloney murine leukemia virus 1 (PIM1) and cellular Myc (c-Myc) was also observed. dBET1 demonstrated a higher level of apoptosis induction compared to JQ-1 in both assays using acute myeloid leukemia (AML) cells and in a murine xenograft model of AML. Furthermore, it demonstrated a positive effect on acute ischemic brain injury by enhancing functional outcomes through the reduction of neuroinflammation, oxidative stress, and the maintenance of blood-brain barrier integrity 106. Two years later, the same group conducted a chemical optimization of dBET1, resulting in the discovery of a highly cell-permeable PROTAC, dBET6 (compound 2) (Figure 4A), with a longer linker length 24. This new drug significantly improved survival in mice with T-cell acute lymphoblastic leukemia (T-ALL) by targeting c-Myc. Notably, the efficacy of dBET6 for the therapeutic management of diverse solid malignancies—such as colon cancer, breast cancer, and melanoma—surpassed that of dBET1 and JQ-1. In addition, dBET6 exhibited a reduction in both the chemoresistance and immune resistance of the cancers it targeted 107. Also, Crews *et al*. developed CRBN-based BRD4 PROTAC, ARV-825 (compound 3) (Figure 4A), that replaced the alkyl linker of dBET1/6 with a polyethylene glycol (PEG) linker 25. Similar to dBET1/6, a flexible PEG linker was chosen due to its high conformational flexibility in order to maximize the chance of the PROTAC binding to both BRD4 and CRBN. The administration of ARV-825 resulted in total BRD4 degradation in cells of Burkitt's lymphoma (BL) (DC_50_ < 1 nM). This degradation led to a sustained reduction in c-Myc levels, an increased impact on inhibiting cell proliferation, and higher levels of apoptosis in BL cells contrasted to small molecule inhibitors. It also potently degraded BRD4 and inhibited cell proliferation in patient-derived secondary AML 108, triple-negative breast cancer (TNBC), ovarian cancer 109, and multiple myeloma (MM) cells 110. Rankovic *et al*. designed phenyl glutarimide (PG) as a novel CRBN binder with improved chemical stability and high ligand efficiency 26. Based on this novel E3 ligand, they designed a novel JQ-1-based BRD4 degrader, SJ995973 (compound 4) (Figure 4A), with a higher efficiency that showed a highly potent antiproliferative efficacy in human AML MV4-11 (IC_50_ = 3 pM). Moreover, it demonstrated the highest level of degradation effectiveness observed in the scientific community, as evidenced by a DC_50_ value of 0.87 nM in MV4-11 cells. The crystal structure of the DDB1∆B-CRBN-dBET23-BRD4BD1 complex (PDB code: 6BN7) was successfully determined by Fischer *et al*. This investigation revealed that changes in the length of the linker and the position of the linkage led to unique binding conformations in the ternary complex 27. Furthermore, the reduction of linker length has been demonstrated to have a notable influence on the degrader's selectivity by restricting the degrader target space and eliminating off-target effects through conformational collisions. This modification has indicated that a new potent PROTAC, ZXH-3-26 (compound 5) (Figure 4A), was identified with a DC_50_ ≈ 5 nM in HEK293T cells. Furthermore, it was found to be isoform-selective, as it did not induce degradation of BRD2/3 at concentrations > 10 μM. Wang *et al*. created a new BRD4 PROTAC, BD-764 (compound 6) (Figure 4A), through the conjugation of QCA276, a pan BET inhibitor previously described, with pomalidomide, a CRBN ligand, using a flexible ethylamino linker 28. Through precise manipulation of the linker, BD-764, a potent and extremely specific BRD4 degrader, led to the acquisition of BD-7148 (compound 7) (Figure 4A) and its more soluble analog, BD-9136 (compound 8) (Figure 4A). These two compounds, which both feature a semi-rigid methylazetidine linker, exhibited degradation potencies in the low-nanomolar range against BRD4 and displayed a remarkable degradation selectivity of over 1,000-fold compared to BRD2 and BRD3. In addition, BD-9136 demonstrated efficient and specific reduction of BRD4 proteins within the tumor tissues of mice while exhibiting no significant impact on BRD2 and BRD3. Additionally, it can also effectively inhibit tumor growth in MV4;11 and MDA-MB-231 xenograft mouse models, without obvious negative effects. Furthermore, it exhibited superior efficacy compared to the corresponding pan BET inhibitor, QCA276. The *in vivo* pharmacodynamic data provided clear evidence that a single administration of of BD-9136 was remarkably efficient in causing a highly efficent and sustained reduction of BRD4 protein levels, specifically within tumor tissues. Furthermore, the impact on BRD2 and BRD3 proteins was either negligible or absent.

Ciulli *et al*. discovered a drug-like VHL ligand, VHL1 (VH032) 111, and then used VHL1 to design a JQ1-based PROTAC MZ1 (compound 9) (Figure 4B), resulting in the targeted degradation of BRD4 in cervical cancer cells, while minimizing the impact on BRD2/3 29. The functionality of MZ1 relies on its interaction with VHL, but this interaction occurs at a concentration that is sufficiently low to impede the stabilization of hypoxia-inducible factor-1α (HIF-1α). MZ1, like JQ-1, induced alterations in the BRD4-dependent genes such as c-Myc, p21, and amphiregulin (AREG), thereby demonstrating its ability to selectively inhibit BRD4. Moreover, the ability to downregulate PD-L1 was confirmed in head and neck squamous cell carcinoma (HNSCC) cell lines. Depending on the ternary complex VHL-MZ1-BRD4's crystal structure, the same group later designed the PROTAC AT1 (compound 10) (Figure 4B) by attaching JQ-1 to the *tert*-Leu group of the VHL ligand 30. AT1 significantly degraded BRD4 (DC_50_ = 10-100 nM, D_max_ > 90%) in all tested cancer cells; however, it showed negligible activity against BRD2 and BRD3, suggesting that AT1 demonstrated a greater degree of selectivity in its ability to deplete BRD4 compared to MZ1.

MDM2 is an E3 ligase that exhibits proficient capability in the p53 tumor suppressor degradation, and inhibitors for it have been widely employed as E3 ligands for the PROTAC-based degradation of a number of oncogenic proteins 112. In 2019, Crews *et al*. created a nutlin-based BRD4 PROTAC A1874 (compound 11) (Figure 4C) that recruited MDM2 31. A1874 was able to degrade BRD4 (DC_50_ = 32 nM) and stabilize p53, thus exhibiting strong anti-proliferative effects in several p53-wild-type cancer cells, such as myeloid leukemia cells.

The degraders that form covalent interactions with either the POI or the E3 ligase have the potential to broaden the range of accessible targets and E3 ligases 113. Additionally, these degraders have the capability to offer supplementary advantages by improving the kinetics of ternary complex formation or introducing enhanced selectivity to the degrader 114. Ward *et al*. employed activity-based protein profiling (ABPP)-based covalent ligand screening methods to discover a covalent ligand for the E3 ubiquitin ligase Ring finger protein 4 (RNF4) 32. Subsequently, they integrated an RNF4 recruiter into bifunctional degraders that were conjugated with the BET inhibitor JQ-1. The RNF4-based PROTAC, CCW 28-3 (compound 12) (Figure 4D), demonstrated a lower level of effectiveness compared to the previously documented JQ1-based degrader MZ1. However, CCW 28-3 was successful in degrading BRD4 through a mechanism reliant on both the proteasome and RNF4. Nomura *et al*. provided evidence for the potential utilization of nimbolide, a naturally occurring anticancer compound, as a covalent binder to facilitate the recruitment of ring finger protein 114 (RNF114) (an E3 ubiquitin ligase). In this study, nimbolide was conjugated with JQ-1, resulting in the synthesis of a novel BRD4 degrader, XH2 (compound 13) (Figure 4D) 33. Upon treatment with XH2 for 12 h, the BRD4 proteasomal degradation was observed in 231MFP breast cancer cells. XH2 demonstrated a reduced level of BRD4 degradation when administered at a concentration of 1 μM, in contrast to concentrations of 0.1 and 0.01 μM. This observation could potentially be attributed to the hook effect. The natural product bardoxolone methyl (CDDO-Me) can establish reversible covalent interactions with cysteine residues located on the KEAP1 E3 ligase 115. In the year 2020, Tong *et al*. conducted a study where they combined CDDO-Me with JQ-1 to create a novel PROTAC that specifically targets BRD4, known as CDDO-JQ-1 (compound 14) (Figure 4D). This newly developed compound was found to effectively stimulate the BRD4 degradation in 231MFP cell line 34. Elimination of the covalent reactive group in CDDO-JQ-1 diminished its ability to degrade BRD4, confirming the importance of the Michael acceptor moiety on CDDO-Me for attracting Kelch-like ECH-associated protein 1 (KEAP1). Recently, Henning *et al*. discovered that the ligand EN106, which possesses a chloroacetamide moiety, exhibits the capacity to establish a covalent bond to the FEM1B E3 ligase 35. NJH-01-106 (compound 15) (Figure 4D), a PROTAC linking EN to JQ-1, was shown to efficiently induce the BRD4 degradation (DC_50_ = 250 nM).

**Figure 5:**
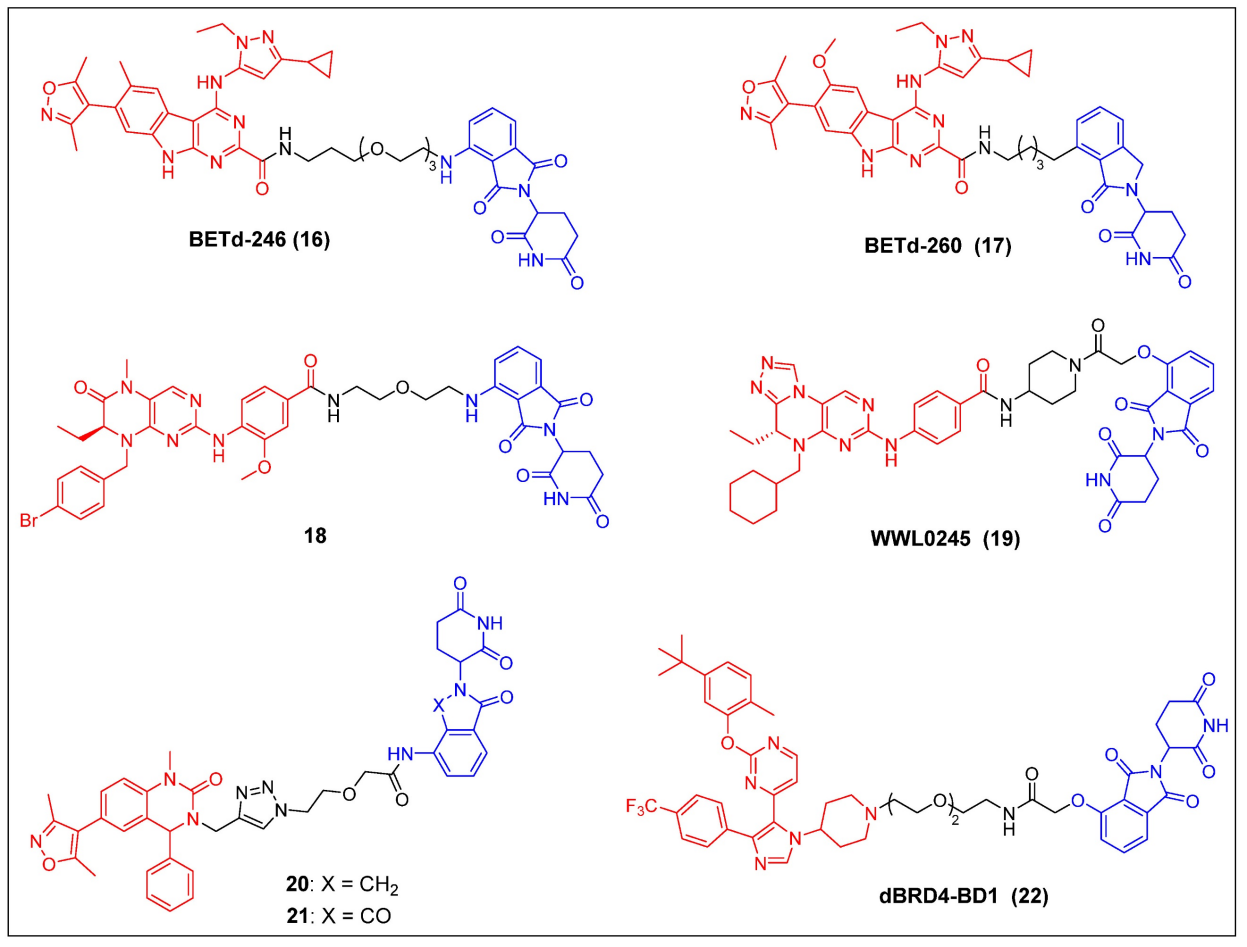
BRD4 PROTACs derived from other BET inhibitors (red for E3 ligand, blue for POI ligand and black for linker).

In recent years, several other potent BET inhibitors besides JQ-1 have also been used as parent BRD4-binders to design PROTACs. In the year 2017, Wang *et al*. presented a BET PROTAC, specifically BETd-246 (compound 16) (Figure 5), which was developed based on their previously reported BET inhibitor BETi-211 37. BETd-246 exhibited a dose-dependent degradation effect on BRD2, BRD3, and BRD4 proteins and demonstrated the capacity to suppress the growth of human TNBC cells at concentrations in the nanomolar range. The application of this degrader to TNBC cells resulted in the observed downregulation of the MCL1 protein, which was found to be time-dependent. Furthermore, in both the Washington University Human-in-Mouse (WHIM) patient-derived xenograft (PDX) and the MDA-MB-453 xenograft mouse models of TNBC, compound BETd-246 successfully reduced BET protein levels in tumors and demonstrated antitumor effects *in vivo*, without overt toxicity to the animals. BETd-260 (also called ZBC260) (compound 17) (Figure 5) was synthesized through a comprehensive optimization process targeting the CRBN-binding moiety of BETd-246 and the linker 36, 37. It was able to effectively stimulate the BRD2/3/4 proteins degradation in the RS4;11 leukemia cells, with DC_50_ values ranging from 30 to 100 pM. The observed degradation was concomitant with significant cleavage of PARP and caspase-3, as well as the significant downregulation of c-Myc. BETd-260 also achieved IC_50_ values of 51 pM and 2.3 nM for inhibiting RS4;11 and MOLM-13 acute leukemia cell growth, respectively. *In vivo* studies have also demonstrated that BETd-260 caused rapid regression of RS4;11 xenograft tumors. Furthermore, BETd-260 was found to induce apoptosis in hepatocellular carcinoma (HCC) xenograft tumors, and it profoundly inhibited the growth of HCC xenograft tumors in mice 116. It also revealed the ability to suppress tumor growth and stem cell-like characteristics by modulating the Wnt/β-catenin signaling pathway in various glioma cell lines, including U-87, U251, H4, and A172 117. Wang *et al*. synthesized another BRD4 degrader, compound 18 (Figure 5), by connecting the potent BRD4 inhibitor BI2536 to the CRBN ligand thalidomide 38. This compound demonstrated significant efficacy in terms of inhibiting BRD4, with an IC_50_ value of 9.4 nM, and effectively suppressed cell proliferation in the BRD4-sensitive RS4;11 leukemia cell line, with an IC_50_ value of 27.6 nM. Furthermore, it was observed that at concentrations of 0.5-1.0 μM, the compound also caused the BRD4 degradation in RS4; 11 cells. Hu *et al*. developed a collection of PROTACs using their previously documented dual inhibitor, WNY0824, which targets both BET and polo-like kinase 1 (PLK1) proteins. This research effort resulted in the identification of a highly effective and isoform-specific BRD4-PROTAC, known as WWL0245 (compound 19) (Figure 5) 39. WWL0245 exhibited significant effectiveness in promoting the BRD4 degradation in prostate cancer cell lines that express AR. It achieved this effect with a DC_50_ value below one nanomolar and a D_max_ value surpassing 99%. Additionally, it exhibited notable selectivity for BRD4, as its ability to induce degradation in other members of the BET family (BRD2, BRD3) and PLK1 was minimal, even at 10 mM. In addition, WWL0245 demonstrated notable inhibitory impacts on the proliferation of AR-positive prostate cancer cell lines that are susceptible to BET inhibitors. In particular, an IC_50_ value of 3 nM was found in MV4-11 cells. Ultimately, it was discovered that the WWL0245 caused an arrest in the cell cycle at the G0/G1 phase and triggered programmed cell death in prostate cancer cells that express AR. This was achieved by decreasing the levels of AR, PSA, and c-Myc proteins and transcriptionally inhibiting genes regulated by AR. Zhang *et al*. have successfully devised a category of dihydroquinazolinone-derived BRD4 PROTACs by integrating a BRD4 inhibitor with a CRBN ligand, specifically lenalidomide or pomalidomide 40. Among them, the lenalidomide-based PROTAC compound 20 (Figure 5) exhibited the highest inhibitory activity against BRD4, with an IC_50_ value of 14.2 nM. The pomalidomide-based PROTAC compound 21 (Figure 5) was the most potent in the group for suppressing the growth of the THP-1 cell line (IC_50_ = 0.81 µM), exhibiting a significantly higher efficacy than compound 20. Mechanistic investigations later revealed that compound 21 effectively induced the BRD4 degradation and suppressed c-Myc. Divakaran *et al*. builds a BRD4 PROTAC, dBRD4-BD1 (compound 22) (Figure 5), by conjugating their previously designed selective BRD4 bromodomain inhibitor, iBRD4-BD1, with a 4-hydroxythalidomide analog using a PEG linker 41. This new PROTAC showed selective and durable degradation of BRD4 (DC_50_ = 280 nM, D_max_ = 77%). Surprisingly, dBRD4-BD1 also upregulated BRD2/3 protein levels at concentrations at which BRD4 was degraded.

**Figure 6:**
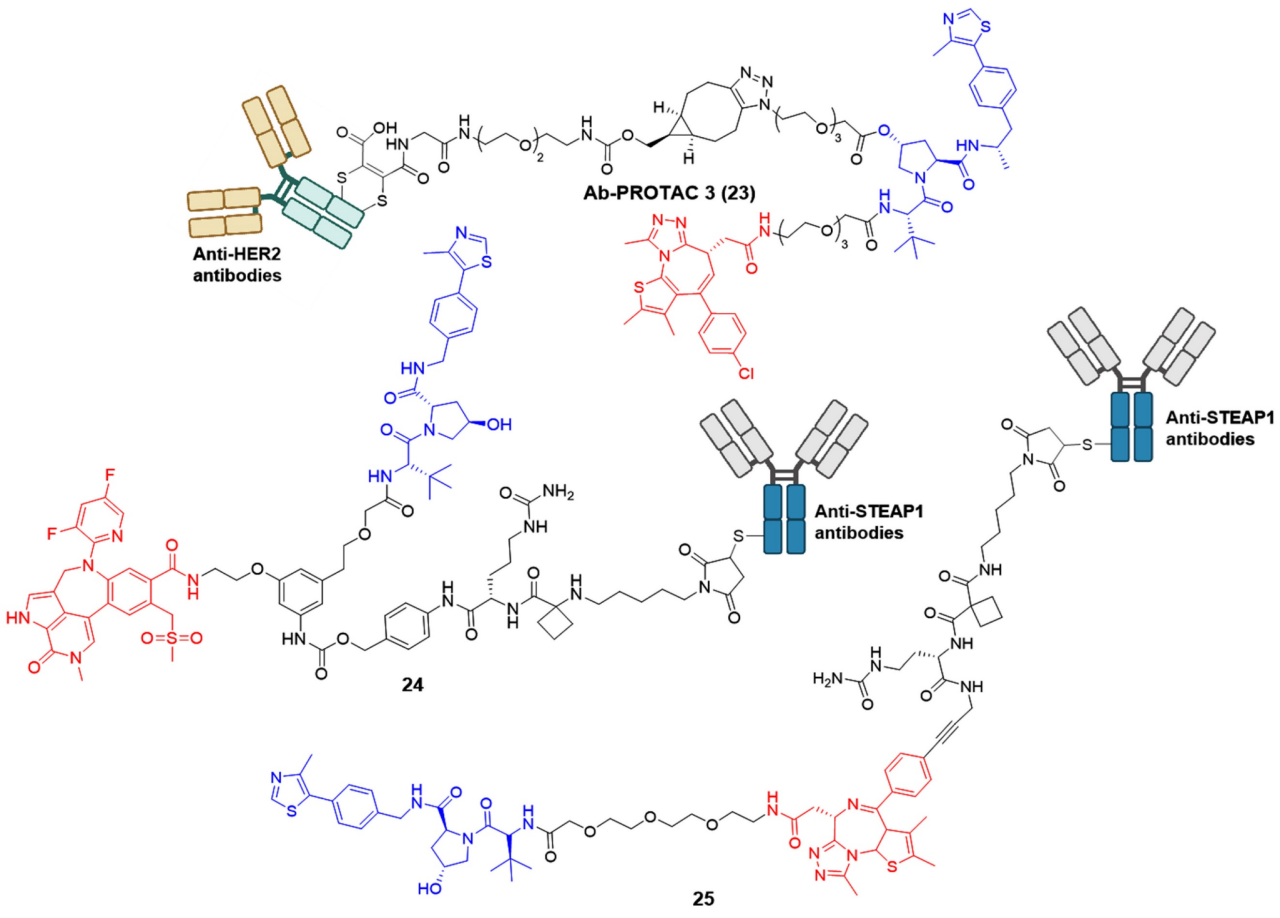
BRD4 Ab-PROTACs (red for E3 ligand, blue for POI ligand and black for linker).

Although many reported PROTACs exhibit high efficiency in degradation, most of them demonstrate limited intrinsic tissue selectivity and cannot distinguish between various cell types. Considering Ab-PROTACs as a prospective alternative could enhance the targeted transport of broad-spectrum PROTACs into specific cell types, aiming for maximal impact on cancer cells with minimal effects on healthy ones 42. In 2020, Tate group reported the conjugation of anti-HER2 monoclonal antibody (mAb) trastuzumab to a BRD4 PROTAC, leading to the identification of Ab-PROTAC 23 (Figure 6) 42. Compound 23 exhibited specific degradation of BRD4 exclusively in HER2-positive breast cancer cell lines SK-BR-3 and BT474, while being rarely internalized to HER2-negative normal cell lines MCF-7 and MDA-MB 231. Upon endocytosis into cancer cells, compound 23 underwent specific internalization and lysosomal trafficking within HER2-positive cells, thereby facilitating the release of the active PROTAC and effectively inducing significant irreversible BRD4 degradation. The majority of PROTACs suffer from a lack of tissue and cell specificity, causing indiscriminate degradation of target proteins in various tissues during *in vivo* applications. Dragovich *et al*. addressed this issue in 2021 by developing a cell-selective PROTAC technology that utilizes Ab-PROTAC conjugates. This innovative approach allows for the targeted degradation of specific proteins within distinct cells, enhancing the selectivity of PROTAC technology at the cellular or tissue level. The authors successfully implemented this technology in developing BRD4 degraders 43, 44. Initially, the conjugate 24 (Figure 6) was designed by tethering a BRD4 degrader MZ1 to an antibody specifically recognizing the six transmembrane epithelial antigen of the prostate 1 (STEAP1) cell surface antigen 43. Compound 24 showed excellent BRD4 degradation potency with a DC_50_ of 0.67 nM in PC-3-S1 cell line. However, its antiproliferative activity was limited (IC_50_ < 790 nM). Subsequent investigations involved modifying the BRD4 degrader's structure and designing a novel set of Ab-PROTAC conjugates 44. Notably, conjugate 25 (Figure 6) demonstrated significant BRD4 protein degradation activity (DC_50_ = 1.4 nM) and displayed effective antiproliferative effects against PC-3-S1 cells (IC_50_ = 29 nM). In addition, conjugate 25 displayed antigen-dependent antitumor activity in both PC-3-S1 and HL-60 xenograft models.

**Figure 7:**
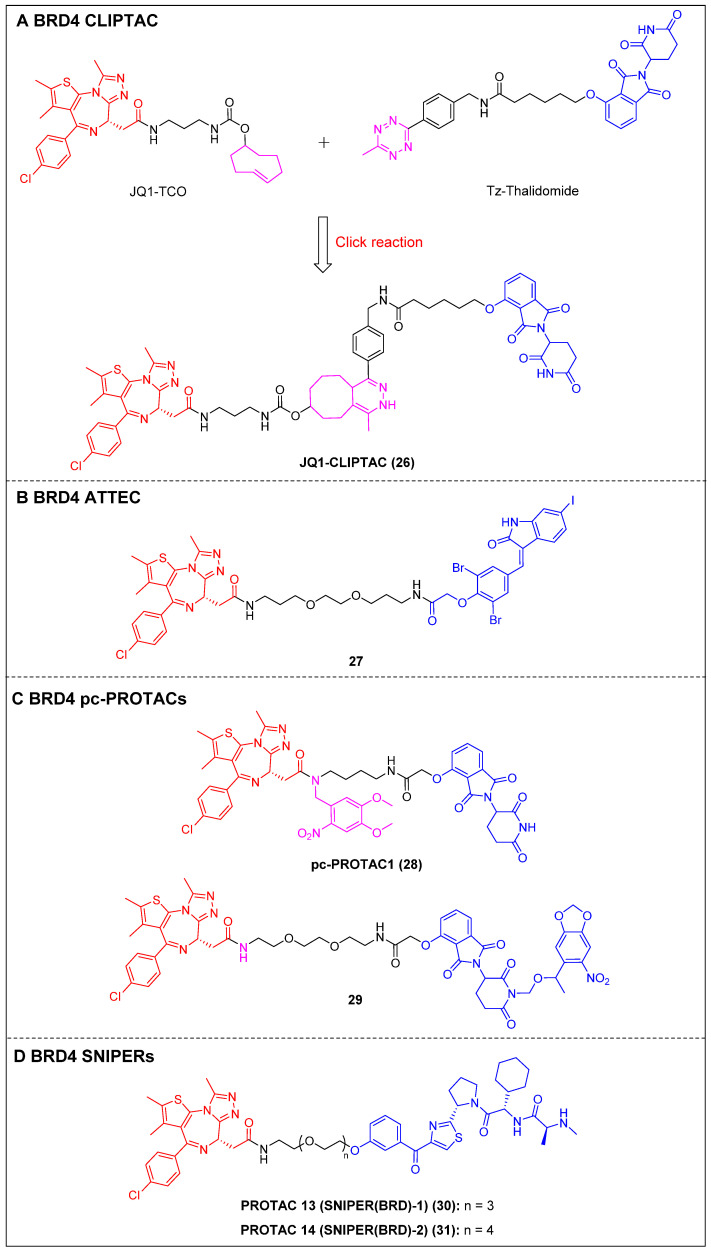
Other BRD4 degraders using emerging degrader technologies (red for E3 ligand, blue for POI ligand and black for linker). (A) BRD4 CLIPAT; (B) BRD4 ATTEC; (C) BRD4 pc-PROTACs; (D) BRD4 SNIPER.

To address the challenges posed by the high molecular weight (MW) and large topological polar surface area (TPSA) of typical small-molecule PROTACs, Lebraud *et al*. introduced a novel approach known as CLIPTACs 15. This strategy aims to mitigate issues related to cell permeability and solubility CLIPTACs can be synthesized via a bio-orthogonal click reaction involving two smaller precursors that are labeled and capable of penetrating the cell membrane. In this investigation, HeLa cells were subjected to treatment with a *trans*-cyclo-octene (TCO)-tagged ligand targeting BRD4 (JQ1-TCO) and a tetrazine-tagged E3 ligase recruiter (Tz-Thalidomide) for a duration of multiple hours, resulting in JQ1-CLIPTAC (compound 26) (Figure 7A) being click-formed intracellularly. There, it successfully degraded BRD4 by recruiting CRBN. More importantly, the epimer of JQ1-TCO that was inactive and the methylated form of thalidomide did not elicit significant degradation, confirming that the noted BRD4 degradation was attributable to the *in situ* JQ1-CLIPTAC formation.

ATTECs function by degrading target proteins via the lysosomal pathway rather than the more common proteasomal one. Compared to the PROTAC technology, ATTECs do not rely on the ubiquitination pathway-mediated degradation, thus avoiding problems associated with insufficient proteasomes and drug resistance caused by targeting E3 ligases 118. ATTEC molecules do not necessitate the use of linkers, possess lower MW, and exhibit favorable membrane permeability. These attributes enhance their compatibility with PK requirements, potentially resulting in improved drug characteristics. In 2021, Ouyang *et al*. demonstrated a class of BRD4 ATTECs by linking the autophagy key protein of the LC3-binding warhead GW5074 to JQ-1 45. Among them, compound 27 (Figure 7B) was identified as the most potent and effective compound, achieving a D_max_ of 92% with a DC_50_ of 0.9 µM in HeLa cells. It was further demonstrated the ability to effectively induce programmed cell death and prolong the G1 phase, thereby demonstrating notable anti-proliferative effects in multiple tumor cells.

By incorporating control elements into PROTACs, they can be activated at specific locations or specific times to exert their effects within cancer cells, thereby conferring selectivity and minimizing potential toxicity. The use of light-induced regulation in biomedicine has gained significant traction in recent years due to its non-invasive nature, rapid efficacy, and high spatial and temporal precision. In 2019, Pan *et al*. developed the first class of pc-PROTACs based on the BRD4 degrader dBET1. A commonly used photo-removable blocking group, 4,5-dimethoxy-2-nitrobenzyl (DMNB), was bonded to the amide nitrogen of JQ1, resulting in the discovery of pc-PROTAC1 (compound 28) (Figure 7C) 46. Notably, pc-PROTAC1 significantly reduced the BRD4 level in live cells upon exposure to ultraviolet (UV) light, which was comparable to the effect of dBET1 degradation. In addition, pc-PROTAC1 exhibited potent antiproliferative activity against Burkitt's lymphoma cells (GI_50_ = 0.4 μΜ), similar to dBET1 (GI_50_ = 0.34 μΜ). Furthermore, uncaged pc-PROTAC1 efficiently degraded BRD4 and inhibited tongue squamous cell carcinoma (TSCC) HN-6 cell growth in zebrafish. In 2020, Deiters *et al*. installed a photocaged group, the 6-nitropiperonyloxymethyl (NPOM) group, at the glutarimide nitrogen to cage the thalidomide moiety 47. Upon irradiation, the generated photocaged BRD4 degrader 29 (Figure 7C) released its parental compound and achieved BRD4 degradation at a micromolar level in HEK293T cells. SNIPERs represent a class of small molecule protein degraders that consist of a combination of the apoptosis inhibitor family known as IAPs and the ligand specific to the POI. The utilization of E3 ligases of IAPs is employed to facilitate the process of ubiquitination and further proteasomal degradation of the POI 119. Unlike other PROTACs that rely on different ubiquitin ligases such as CRBN and VHL, SNIPERs can degrade cIAPI and thus influence the degradation of target proteins, but this potential limitation may be addressed through the selection and optimization of IAP inhibitors.

Currently, more than 20 proteins have been documented as being subject to degradation by SNIPERs, highlighting their promising future in the field 120. In 2019, Ohoka *et al*. created two BRD4 PROTACs, SNIPER(BRD)-1 (compound 30) (Figure 7D) and SNIPER(BRD)-2 (compound 31) (Figure 7D), by combining the BET inhibitor JQ-1 with an IAP antagonist LCL-161 derivative 48. Both SNIPER(BRD)-1 and SNIPER(BRD)-2 caused significant reductions in BRD4 protein levels. SNIPER(BRD)-1 also effectively induced the degradation of cellular inhibitors of apoptosis protein 1 (cIAP1) and X-linked inhibitor of apoptosis protein (XIAP). Mechanistic studies revealed that the degradation of cIAP1 was initiated through the IAP antagonist module of SNIPER(BRD)-1, independent of the intrinsic target protein. The degradation process was initiated by the auto-ubiquitination of cIAP1. In contrast, the degradation of XIAP and BRD4 induced by conventional SNIPERs necessitated the formation of a ternary complex.

#### 2.1.3 BRD2

**Figure 8:**
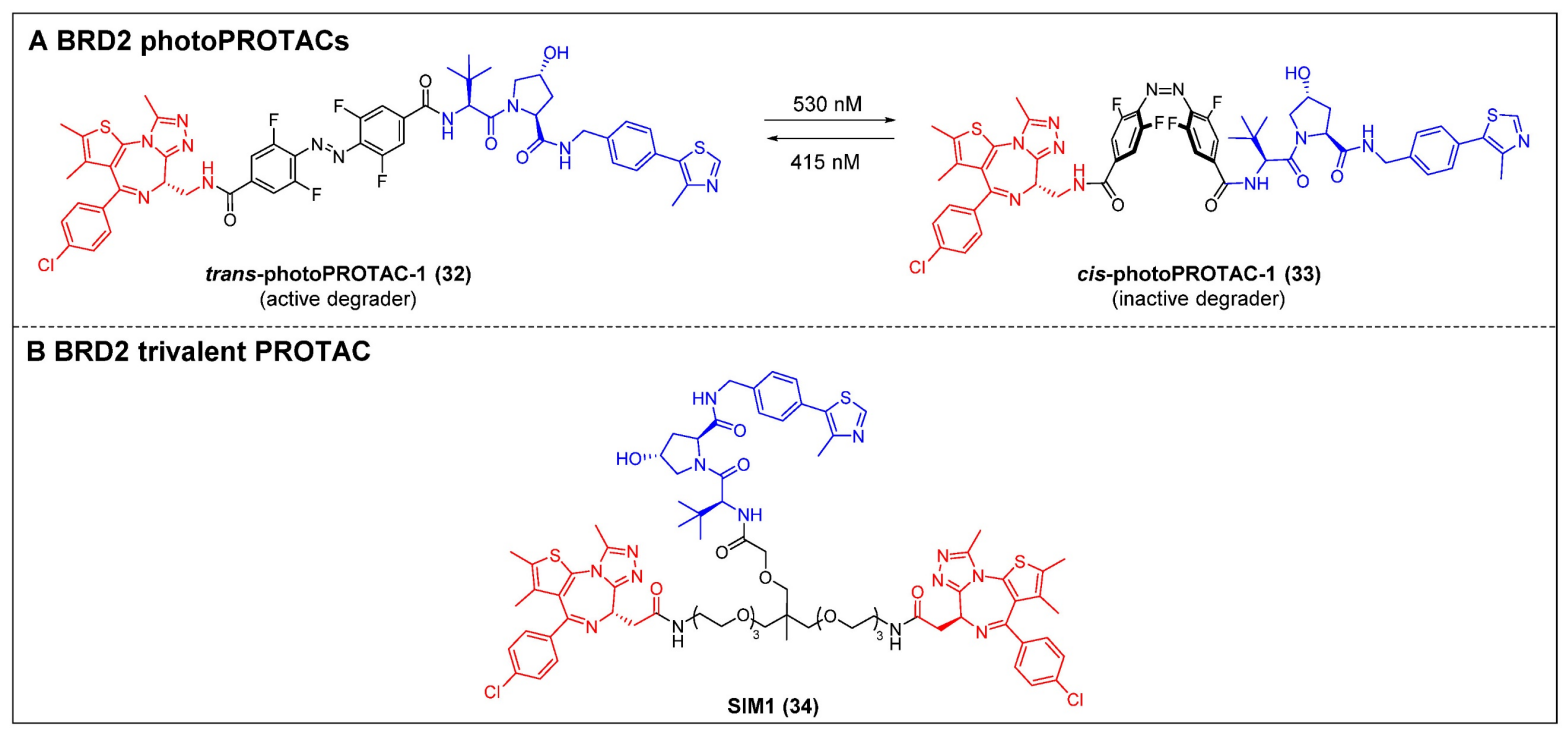
BRD2 degraders (red for E3 ligand, blue for POI ligand and black for linker). (A) BRD2-photoPROTAC; (B) BRD2-trivalent PROTAC.

The photo-switchability PROTACs provide a mechanism for reversible modulation of targeted protein degradation, allowing for spatiotemporal control 49. In 2019, Crews *et al*. introduced the first photoPROTAC by substituting the oligoether linker in ARV-771 with a photo-switchable *ortho*-F4-azobenzene linker 49. *Trans*-photoPROTAC-1 (compound 32) (Figure 8A) effectively eliminated BRD2 in Ramos cells under 415 nm irradiation; in contrast, the irradiation of the PROTAC with 530 nm light did not result in any apparent degradation, which can be attributed to the *trans*-to-*cis* transformation induced by the light. Although ARV-771 has the capability to degrade both BRD4 and BRD2, there was no significant degradation of BRD4 observed in both *trans*- and *cis*-photoPROTAC-1 (compound 33) (Figure 8A). Moreover, the persistence of the photostationary state (PSS) of photoPROTAC is attributed to the bi-stable nature of the *ortho*-F4-azobenzene moiety, allowing it to maintain its state even without continuous irradiation. The present innovative methodology facilitates a reversible activation and deactivation mechanism for protein degradation, which is compatible with the intracellular milieu, making it a potentially valuable tool for investigating poorly understood protein signaling pathways.

Enhancing the binding valency of PROTACs towards a specific target represents a valuable approach to enhance both the efficiency of degradation and the selectivity towards the intended target. In the year 2021, Ciulli *et al*. developed a trivalent PROTAC denoted as SIM1 (compound 34) (Figure 8B). This was achieved by combining a bivalent BET inhibitor with a VHL ligand, connected through a branched linker that is exposed to the solvent 50. SIM1 demonstrated a potent and persistent reduction in the levels of BRD2/3/4 and c-Myc in BET-sensitive human HEK293 cells at picomolar concentrations. This indicates that SIM1 exhibits significantly greater efficacy compared to the bivalent PROTAC molecules MZ1 and ARV-771. In contrast to MZ1, which demonstrated a preference for degrading BRD4, SIM1 displayed a notable selectivity towards BRD2 (DC_50_ = 1.1 nM), despite its potent inhibitory activity against BRD4. The viability of MV4-11 cells was effectively suppressed by SIM1, and apoptosis was induced in the prostate cancer cell line 22RV1. Mechanistically, SIM1 was found to engage the BD2 and BD1 bromodomains of BRD4 to form a ternary complex with VHL and BRD4, thus elevating target binding valency and prolonging specific molecular interactions with the target. In contrast to bivalent compounds, SIM1 demonstrated superior cell permeability and a notably favorable PK profile, despite its substantial molecular weight.

#### 2.1.4 BRD9

**Figure 9:**
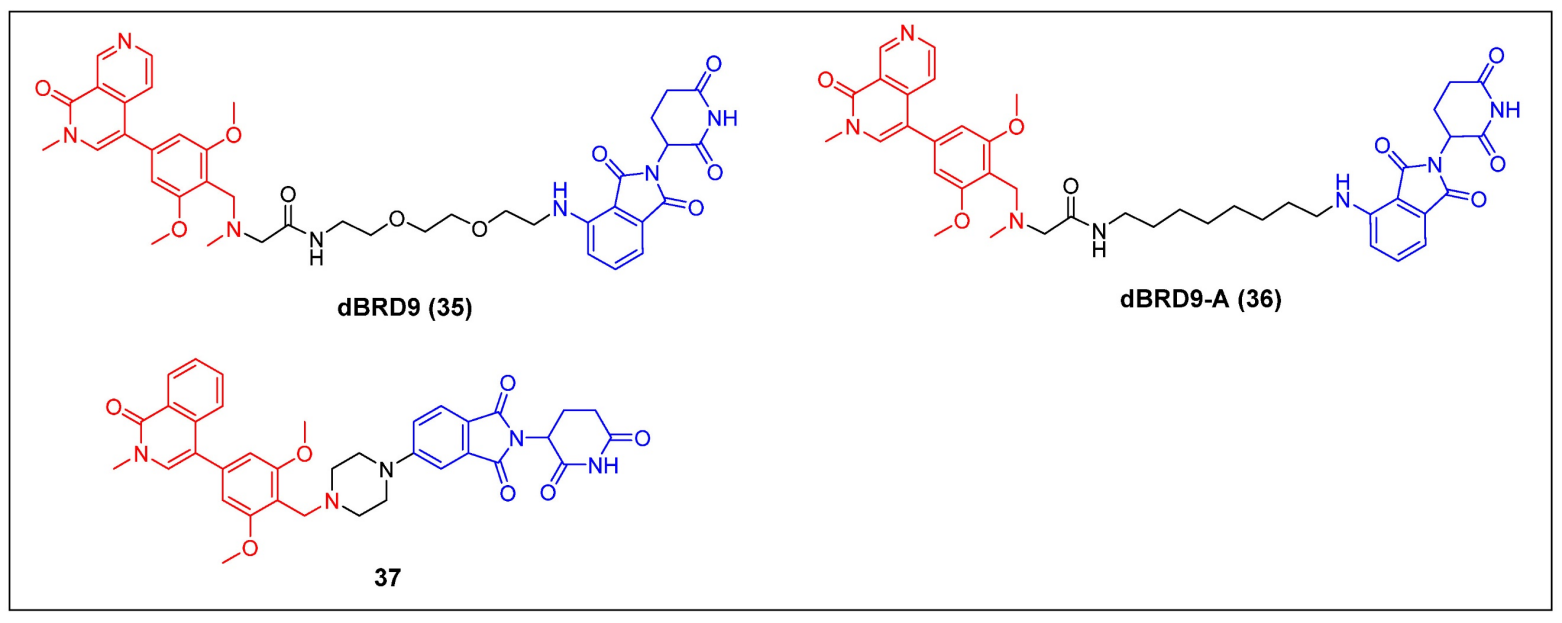
BRD9 PROTACs (red for E3 ligand, blue for POI ligand and black for linker).

Bradner *et al*. reported the first BRD9 degrader, dBRD9 (compound 35) (Figure 9), by linking a highly selective BRD9 inhibitor, BI-7273, and the CRBN ligand pomalidomide, using a PEG-based linker 51. The compound demonstrated effective degradation of BRD9 with a DC_50_ value of 50 nM and an IC_50_ value of 104 nM. Additionally, it displayed stronger inhibitory effects on cell proliferation in the human AML MOLM-13 cell line compared to its parental ligand, BI-7273. Furthermore, it demonstrated cytotoxic effects on the EOL‐1 AML eosinophilic and A‐204 malignant rod‐like cell lines. Following this, the same group identified dBRD9-A (compound 36) (Figure 9) using a more lipophilic alkyl linker compared with its close analogue dBRD9 52. dBRD9-A demonstrated a significant ability to degrade BRD9 in synovial sarcoma cell lines HSSYII and SYO1. Moreover, dBRD9-A decreased the association of BRD9 with chromatin, leading to enhanced anti-proliferative effects. Additionally, dBRD9-A inhibited synovial sarcoma tumor proregression and oncogenic transcription. Importantly, dBRD9-A-treated mice exhibited no significant adverse effects, maintaining normal body weights and blood counts.

Recently, Zhang *et al*. described a new orally bioavailable BRD9 PROTAC 37 (Figure 9) based on BRD9 inhibitor BI-7271 and CRBN ligand 53. Compound 37 demonsstrated potent activity in degrading BRD9 (DC_50_ =1.02 nM), while not causing BRD4 or BRD7 degradation. Besides, C6 also showed strong anti-proliferative effects against AML cell line MV4-11 (IC_50_ = 3.69 nM). Additionally, compound 37 exhibited remarkable oral activity.

#### 2.1.5 Multi-target BET proteins

**Figure 10:**
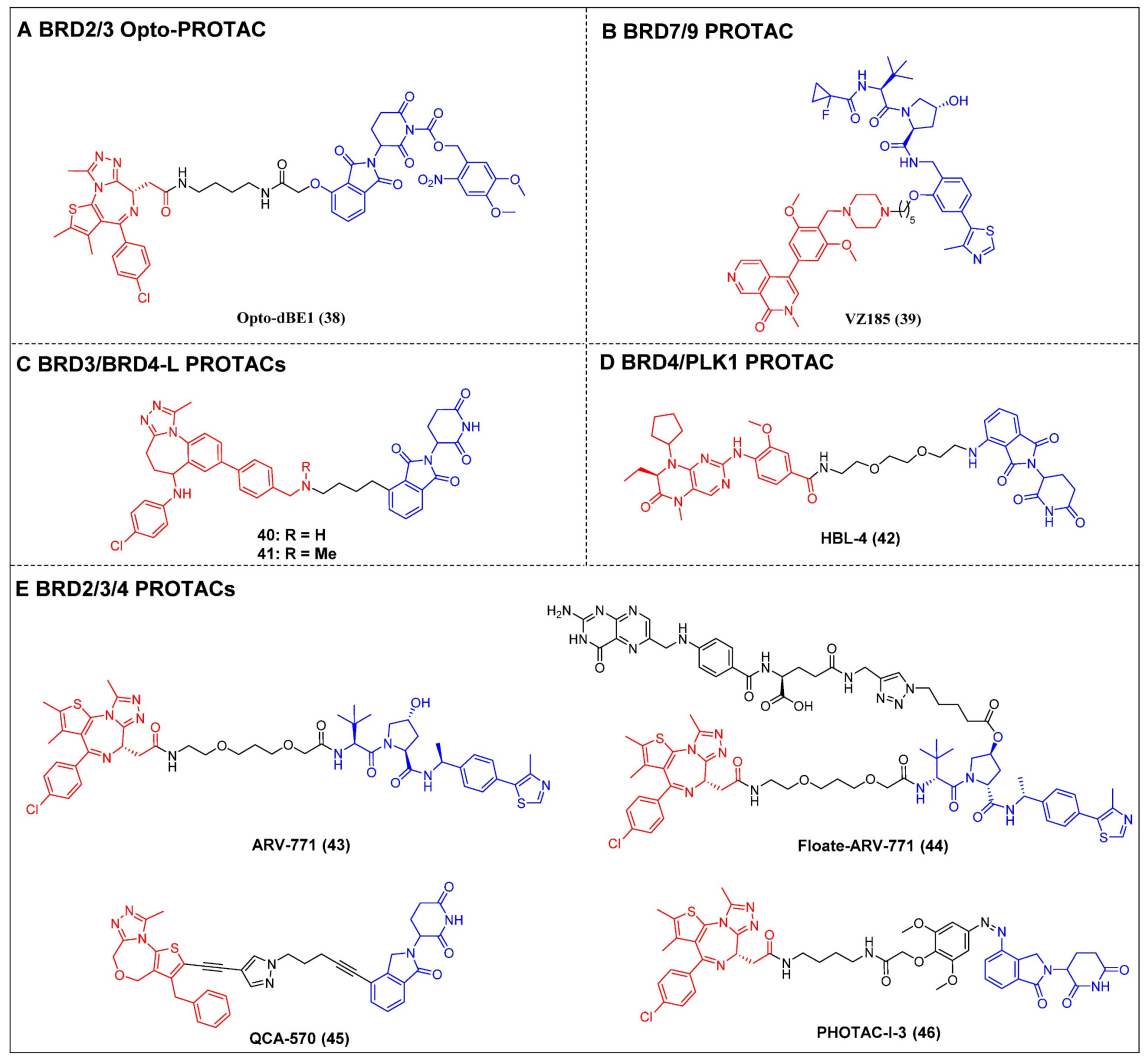
Multi-target BET degraders (red for E3 ligand, blue for POI ligand and black for linker). (A) BRD2/3 Opto-PROTAC; (B) BRD7/9 PROTAC; (C) BRD3/4-L PROTACs; (D) BRD4/PLK1 PROTAC; (E) BRD2/3/4 PROTACs.

Opto-PROTAC was invented in 2020 by Jin *et al*. The BRD4-targeting PROTAC opto-dBET1 (compound 38) (Figure 10A) was designed by adding a photolabile caging group to pomalidomide to impede the recruitment of ubiquitin to CRBN 121. The BRD2/3 degradation in a spatiotemporal manner was demonstrated through biochemical and biological evaluations, specifically under the influence of light at 365 nm.

BRG1/BRM-associated factor (BAF) and polybromo-associated BAF (PBAF) are two variants of the SWI/SNF complex. They are involved in regulating gene expression, DNA replication, and DNA repair 122. BRD9 and its paralog bromodomain-containing protein 7 (BRD7) are subunits of the BAF and PBAF chromatin remodeling complexes, respectively 123. A selective dual BRD7 and BRD9 degrader, VZ185 (compound 39) (Figure 10B), that uses BI-7273 as a warhead was described by Ciulli *et al*. in 2019 124. In RI-1 cells, VZ185 demonstrated efficient degradation of both BRD9 and BRD7 proteins, exhibiting DC_50_ values of 1.8 and 4.5 nM, respectively. Furthermore, it demonstrated inhibitory effects on the growth of pretreated EOL‐1 acute myeloid eosinophilic leukemia (EC_50_ = 3 nM) and A‐204 malignant rhabdoid tumor (EC_50_ = 40 nM) cell lines. The *in vitro* PK data also indicated an increased stability of VZ185 in microsomes and plasma obtained from human and mouse subjects.

Recently, Zhao *et al*. introduced a novel PROTAC (compound 40) (Figure 10C) that utilized a selective inhibitor of BET BD2s, BY27, in combination with lenalidomide 125. Compound 40 demonstrated the ability to selectively degrade BRD3 and BRD4-L proteins while leaving BRD2 and BRD4-S unaffected in various cell lines, including MM.1S, HGC-27, MCF-7, A549, HeLa, and HepG2. In the MM.1S mouse xenograft model, a refined PROTAC (compound 41) (Figure 10C) facilitated BRD3 and BRD4-L degradation* in vivo*, resulting in a substantial antitumor effect.

Lu *et al*. have reported on a dual degrader of BRD4 and PLK1 utilizing CRBN, which are both considered promising therapeutic targets for AML, HBL-4 (compound 42) (Figure 10D), based on the dual-target inhibitor BI2536 126. The administration of HBL-4 resulted in rapid, effective, and sustained BRD4 and PLK1 degradation in MV4-11 cells, both *in vitro* and* in vivo*. Additionally, HBL-4 exhibited excellent inhibitory effects on cell proliferation and induced degradation of BRD4 and PLK1 in human acute leukemia cells MOLM-13 and KG1. It also suppressed c-Myc levels more effectively than its parental inhibitor BI2536, leading to a heightened induction of apoptotic activity in MV4-11 cells. HBL-4 exhibited significantly enhanced efficacy in a tumor xenograft model of MV4-11 when compared to the BI2536.

Raina *et al*. described a VHL-based pan-BET degrader, ARV-771 (compound 43) (Figure 10E) 54. ARV-771 efficient degradation of BRD2/3/4 in the 22Rv1 cell line, exhibiting a DC_50_ value of < 5 nM. Compared to BET inhibitors, in the castration-resistant prostate cancer (CRPC) xenograft model, ARV-771 could concurrently suppress the expression of the AR protein and impede the transmission of the AR signal. Consequently, this dual action results in the regression of tumors. Additionally, this study represents the initial documentation of a BET degrader exhibiting *in vivo* efficacy in the context of solid tumor malignancy. Subsequent investigations revealed that the administration of ARV-771 exhibited a more pronounced decrease in leukemia load and a further enhancement in survival rates among NOD-scid IL2rγnull (NSG) mice that were engrafted with luciferase-labelled HEL92.1.7 cells, in comparison to the administration of the BET inhibitor OXT015 108. ARV‐771 significantly suppressed tumor growth *in vivo* and improved the survival of mantle cell lymphoma (MCL)-cell-engrafted nude mice, and co-treatment with ARV-771 and ibrutinib or venetoclax (BCL2‐antagonists) or palbociclib (CDK4/6 inhibitor) synergistically induced apoptosis in MCL cells 127. In order to mitigate the potential adverse effects resulting from off-tissue on-target degradation, Wei *et al*. introduced folate to the hydroxyl group of VHL 128. This researchers successfully developed a folate-caged PROTAC, denoted as Folate-ARV-771 (compound 44) (Figure 10E). Folate-ARV-771 demonstrated efficient BRD2/3/4 protein degradations in cancer cells through a mechanism that is dependent on folate receptor alpha (FOLR1), but not in normal non-cancerous ones. Nevertheless, the introduction of the folate group conjugation resulted in a notable elevation in the MW of the PROTAC, surpassing 1,000 Da. This could potentially undermine its ability to be absorbed orally and its PK properties. Wang *et al*. developed a BET degrader, QCA570 (compound 45) (Figure 10E), that simultaneously caused the BRD2, 3, and 4 degradations in human leukemia cell lines and showed picomolar potency against MV4-11, MOLM-13, and RS4-11 human acute leukemia cell lines 55. Notably, QCA570 demonstrated a full and long-lasting reduction in tumor size at well-tolerated dosing schedules without severe toxicity in MV4-11 and RS4;11 acute leukemia xenograft models. Furthermore, according to reports, there was an effective inhibition of the growth of non-small cell lung cancer (NSCLC) cells in humans and showed a synergistic effect with osimertinib for suppressing osimertinib-resistant EGFR-mutant NSCLC cells both* in vitro* and *in vivo* 13. QCA570 also potently induced BRD4 degradation (DC_50_ ≈ 1 nM) in several breast cancer cell lines at nanomolar concentrations 129. The findings demonstrated a reduction in EZH2 and c-Myc levels via both transcriptional repression and protein degradation. Furthermore, the degrader exhibited a notable capacity to induce cellular apoptosis and cycle arrest in breast cancer cells, as well as demonstrated significant antiproliferative effects against breast cancer cells. Recently, Reynders *et al*. have developed an additional series of PROTACs that target BRD2-4 proteins, which have been termed PHOtochemically TArgeting Chimeras (PHOTACs) 56. Among them, PROTAC-I-3 (compound 46) (Figure 10E) has been identified as a highly potent degrader of BRD2/4. PROTAC-I-3 features the integration of an azo-double bond directly onto its pomalidomide motif. Upon irradiation with 390 nm UV light, the activation of PROTAC-I-3 resulted in a faster recovery of BRD2 levels in RS4;11 cells treated with 525 nm irradiation contrasted to control cells kept in the dark. A reduction in the levels of BRD4 was observed upon exposure to PHOTAC-I-3, particularly within the concentration range of 100 nM to 3 µM, under irradiation with 390 nm light. However, no such decrease in BRD4 levels was observed in the absence of light. In a similar manner, the levels of BRD3 were significantly diminished following exposure to PHOTAC-I-3 at varying concentrations (ranging from 100 nM to 3 µM) under UV light irradiation but not under conditions of darkness.

### 2.2 Non‑BET proteins

#### 2.2.1 CBP/p300

Lysine acetyltransferases (KATs) refer to a class of enzymatic proteins that facilitate the acetylation of lysine residues, which occurs in both histone and non-histone proteins. The proteins primarily encompass the p300/CREB-binding protein (CBP), the GNAT family (GCN5 and PCAF), and the M oz, Y bf2/Sas3, S as2, and T ip60 (MYST) family 130. The proteins CBP and p300 exhibit a strong correlation, as evidenced by their 96% sequence similarity in BRDs. These proteins play a crucial role in maintaining gene expression patterns by facilitating chromatin lysine acetylation and transcriptional regulation. The proteins CBP and p300 are involved in promoting cancer cell proliferation, survival, tumorigenesis, metastasis, immune evasion, and drug resistance 131. Although the development of small-molecule CBP/p300 histone acetyltransferase inhibitors has sparked broad interest with regard to cancer treatment, inhibiting a single domain of one of these targets cannot completely ablate its function, as p300/CBP is comprised of eight discrete functional domains 132. Thus, CBP/p300 protein degraders are currently being recognized as potentially effective and innovative anticancer agents for clinical application, by fully depleting them, the p300/CBP-mediated enhancer activity is ablated.

**Figure 11:**
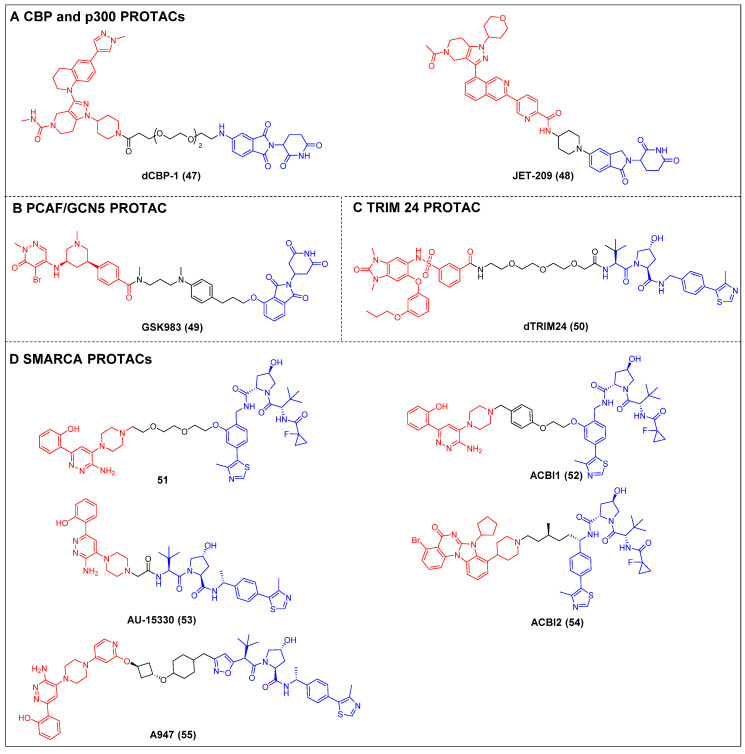
Non-BET PROTACs (red for E3 ligand, blue for POI ligand and black for linker). (A) CBP and p300 PROTACs; (B) PCAF/GCN5 PROTACs; (C) TRIM24 PROTACs; (D) SMARCA PROTACs.

Ott *et al*. created the first-in-class CBP and p300 degrader, dCBP-1 (compound 47) (Figure 11A), based on the CRBN ligand 57. It showed potent antiproliferative activity in MM cells and demonstrated augmented impacts on the expression patterns of the c-Myc gene, mechanisms that inhibit cell proliferation, and the structure of chromatin in MM. The utility of dCBP-1 as a potent acetyltransferase inhibitor has been demonstrated in its application for investigating the underlying mechanisms governing enhancer activity in both healthy and malignant cells. Wang *et al*. developed and produced a novel and highly effective PROTAC degrader called JET-209 (compound 48) (Figure 11A). This degrader specifically targets the proteins CBP/p300 and was designed using the BRD inhibitor GNE-207 as a foundation 133. JET-209 effectively degraded CBP (DC_50_ = 0.05 nM) and p300 (DC_50_ = 0.2 nM), with a D_max_ of >95% for both proteins, in RS4;11 leukemia cells. It also exhibited sub-nanomolar to low-nanomolar potency for inhibiting cell growth in MV4, 11, HL-60, and MOLM-13 cell lines—surpassing the potency of CBP/p300 BRD and catalytic domain inhibitors. Additionally, JET-209 demonstrated its efficacy in suppressing tumor growth in xenograft tumors.

#### 2.2.2 PCAF/GCN

P300/CBP-associated factor (PCAF) and general control nonderepressible 5 (GCN5), exhibit high similarity and possess multiple domains. These domains include an acetyltransferase domain and a BRD 134. PCAF is capable of producing certain inflammatory factors 135. Nevertheless, chemical inhibition specifically aimed at the PCAF/GCN5 BRD did not prove to be effective in reducing the inflammatory response exhibited by immune cells lacking PCAF.

Tough *et al*. created the first CRBN-PROTAC GSK983 (compound 49) (Figure 11B), which specifically targeted PCAF and GCN 136. At nanomolar concentrations, GSK983 potenly induced PCAF/GCN5 degradation in acute monocytic leukemia THP1 cells. Moreover, the degradation of GCN5/PCAF PCAF/GCN5 by GSK983 resulted in the downregulation of several inflammatory cytokines in lipopolysaccharide (LPS)-stimulated macrophages and dendritic cells (DCs), which illustrates the superiority of PCAF/GCN-targeting PROTACs over PCAF/GCN BRD inhibitors in their ability to mitigate inflammation.

#### 2.2.3 TRIM24

Tripartite motif-containing 24 (TRIM24), alternatively referred to as transcriptional intermediary factor 1α (TIF1α), is a constituent of the TRIM protein family that encompasses more than 70 identified members, which play a function in the transcriptional regulation of the AR and additional nuclear receptors 137. Elevated levels of TRIM24 have been found to be correlated with the developing cancer and the progressing of disease in various types of cancer.

Bradner *et al*. developed a TRIM24-targeting PROTAC, dTRIM24 (compound 50) (Figure 11C), by conjugating a TRIM24 BRD inhibitor IACS-9571 and to a VHL ligand 138. This PROTAC caused the effective and selective TRIM24 degradation and significantly upregulated tumor suppressor genes in AML cells. By employing dTRIM24 to examine the role of TRIM24, an analysis was conducted to determine the impact of TRIM24 loss on chromatin localization and gene regulation across the entire genome. The comparative investigation of TRIM24 degradation and BRD inhibition demonstrated a heightened antiproliferative effect resulting from degradation.

#### 2.2.4 SMARCA

The multi-subunit switch/sucrose non-fermentable (SWI/SNF) complex formed by ATP-dependent helicases (ATPases), SMARCA2 or SMARCA4, facilitates chromatin remodeling to regulate a number of cellular processes 139. SMARCA4 is necessary for maintaining the oncogenic transcriptional regime and driving proliferation in AML 140. The potential therapeutic approach of targeted inhibition of SMARCA2 has been put forth as a viable treatment strategy for cancers harboring SMARCA4 mutations, and proliferation of the SMARCA4 mutant xenograft model was effectively inhibited by allosteric inhibitors of SMARCA2/4 141. Previous studies have documented the existence of small molecule ligands that specifically target the BRD regions of SMARCA2/4 (SMARCA2/4BD), to date, attempts to replicate these anti-proliferative effects have been unsuccessful, as the presence of an intact BRD of SMARCA2 is not necessary for the maintenance of proliferation 142. Therefore, PROTACs that selectively target the non-functional BRD of SMARCA2/4 presents a potential avenue for exploiting the susceptibilities of cancer cells that rely on SMARCA2 or SMARCA4 for their survival.

Cilla *et al*. devised a collection of degraders that selectively act on SMARCA2/4 58. This was achieved by covalently attaching a BRD ligand and a VHL ligand to PEG linkers of different lengths. Prior to this, the researchers determined the crystal structure of the BD inhibitor in complex with SMARCA2BD. This structural analysis enabled the identification of a suitable site for linker attachment. The degrader 51 (Figure 11D) demonstrated efficacy in promoting the moderate SMARCA2/4 degradation within MV4-11 cells, resulting in a D_max_ of approximately 65-70%. In this study, they utilized the high-resolution ternary complex crystal structures of SMARCA2BD-40-VCB as a guiding framework; the research team developed a more potent SMARCA2/4 degrader, ACBl1 (compound 52) (Figure 11D), which exhibited enhancements in ternary complex cooperativity and affinity, along with improved PROTAC permeability. ACBI1 effectively induced the swift, specific, and total degradation of SMARCA2, SMARCA4, and PBRM1 targets within MV-4-11 cancer cells. The DC_50_ values for SMARCA2 and SMARCA4 were determined to be 6 nM and 11 nM, respectively. It was demonstrated that this degrader exhibited antiproliferative effects in AML cell lines, whereas the original inhibitor did not exhibit any significant reduction in cell viability. Chinnaiyan *et al*. established a connection between the identical BRD ligand and VHL. Additionally, they formulated a new degrader, AU-15330 (compound 53) (Figure 11D), specifically targeting SMARCA2 and SMARCA4 59. AU-15330 exhibited the capacity to cause the SMARCA2 and SMARCA4 proteins degradation in HEK293 and HeLa cell lines. Additionally, it exhibited a certain level of degradation activity towards PBRM1. Additionally, it elicited a strong suppression of tumor growth in xenograft models of prostate cancer and demonstrated a synergistic effect when combined with the AR antagonist enzalutamide. Notably, it even resulted in disease remission in models of CRPC without any observed toxic effects. A class of orally bioavailable VHL-recruiting degraders was released by Boehringer Ingelheim Pharma GmbH & Co KG and Ciulli *et al* 60. The researchers employed ternary co-crystal structures as a guiding framework to synthesize a potent and pharmacokinetically favorable PROTAC, ACBI2 (compound 54) (Figure 11D). The study successfully demonstrated the targeted degradation of SMARCA2 over SMARCA4 and observed the therapeutic effectiveness in cancer models with SMARCA4 deficiency. Genetech and Arvinas created a highly effective and reasonably specific VHL-based SMARCA2-targeting PROTAC known as A947 (compound 55) (Figure 11D) 143. A947 exhibited augmented growth inhibitory impacts in SMARCA4^mut^ non-small cell lung cancer (NSCLC) models, as compared to wild-type models, without significant tolerability concerns. It also potently degraded SMARCA2 in SW1573 cells, with a DC_50_ of 39 pM and a D_max_ of 96% at 10 nM, exhibiting high selectivity for degrading SMARCA2 over SMARCA4, with no off-target effects.

## 3. Histone acetylation-/deacetylation-related targets

### 3.1 HDAC family

HDACs play a crucial role in the epigenetic modification process by catalyzing the removal of acetyl groups from lysine residues on histones. This enzymatic activity results in chromatin condensation, ultimately leading to the repression of gene transcription. Additionally, HDACs exhibit a strong affinity for negatively charged DNA, enabling them to tightly bind to it 144. Several members of the HDAC family have the ability to interact with various cellular non-histone proteins, such as DNA-binding proteins, transcriptional co-regulators, chaperone proteins, and structural proteins 145. A total of 18 human HDACs have been discovered, with 11 belonging to the category of zinc-dependent enzymes (HDAC1-11) and the remaining seven falling under the classification of nicotinamide adenine dinucleotide (NAD)-dependent sirtuins (SIRT1-7) 146. Certain HDAC inhibitors, such as vorinostat or panobinostat, which are hydroxamic acid derivatives, have been granted approval for the therapeutic management of hematologic malignancies, but their isoform selectivity is limited 147. Thus, PROTACs offer a promising alternative for addressing the limitations of selectivity in inhibitors that aim to selectively target the enzymatic and non-enzymatic (scaffolding) functions of HDAC.

#### 3.1.1 HDAC6

**Figure 12:**
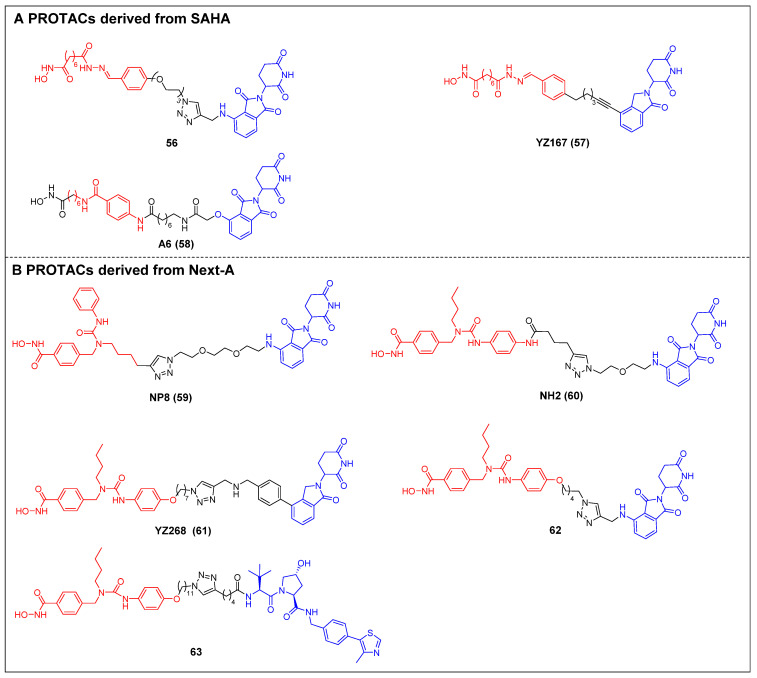
HDAC6 PROTACs (red for E3 ligand, blue for POI ligand and black for linker). PROTACs derived from SAHA (A) and Next-A (B).

The first selective HDAC6 PROTACs were documented by Yang *et al*., who achieved this by linking a pan-HDAC inhibitor, SAHA, with a CRBN ligand resembling thalidomide 61. Among them, compound 56 (Figure 12A) exhibited the highest potency as an HDAC6 degrader in MCF-7 cells, with a DC_50_ value of 34 nM. Compound 56 has the potential to elevate the acetylation level of histone H3 at high concentrations, suggesting its ability to impede the activity of class I HDACs. Following this, two novel SAHA-based CRBN recruiting HDAC6 PROTACs, YZ167 (compound 57) 62 and A6 (compound 58) 63 (Figure 12A), were also reported. YZ167 achieved efficient HDAC6 degradation in MM.1S cells, with a DC_50_ of 1.94 nM. Similarly, A6 degraded HDAC6 effectively, with a DC_50_ of 3.5 nM in HL-60 cells and no off-target effects on HDAC1 and HDAC4. Furthermore, it exhibited a notable antiproliferative effect by triggering apoptosis in cell lines of myeloid leukemia.

Rao *et al*. constructed a series of HDAC6 degraders by conjugating an HDAC6‐selective inhibitor, Next-A, with the CRBN ligand 64. Among them, the PROTAC with the highest level of activity, NP8 (compound 59) (Figure 12B), successfully facilitated the HDAC6 degradation (DC_50_ = 3.8 nM) in the MM.1S cell line. The same group then proceeded to develop a novel HDAC6 PROTAC, NH2 (compound 60) (Figure 12B), wherein pomalidomide was introduced at the benzene ring of Next-A 65. NH2 exhibited a comparable efficiency for degrading HDAC6 (DC_50_ = 3.2 nM) compared to NP8. Two other HDAC6 degraders, YZ268 (compound 61) (Figure 12B) 62 and compound 62 (Figure 12B) 66, with different triazole-containing linkers that were structurally similar to NH2, were also reported by two other research groups. YZ268 exhibited selective degradation activity toward HDAC6 without affecting the neo-substrates IKZFs and GSPT1. Meanwhile, compound 62 showed a significant potency for degrading HDAC6, with a DC_50_ of 1.64 nM and an excellent antiproliferative effect (EC_50_ = 74.9 nM) against MM. However, compound 62 also induced the degradation of undesired neo-substrates, such as IKZF1/3, due to the binding of its pomalidomide component to CRBN. Similarly, Tang *et al*. developed the first VHL-recruiting cell-permeable HDAC6-selective degraders based on Next-A 67. Among them, compound 63 (Figure 12B) exhibited the highest level of degradation activity in human MM1S cells. It demonstrated a DC_50_ value of 7.1 nM and achieved a D_max_ of 90%. The mechanistic studies provided evidence that the targeting of HDAC6 by compound 63 resulted in proteasomal degradation. However, no degradation of IKZF1/3 was observed in this context. Notably, it also showed excellent selectivity over other HDACs. In the 4935 mouse cell line, compound 63 proved effective for inducing HDAC6 degradation, with a DC_50_ of 4.3 nM and a D_max_ of 57%.

#### 3.1.2 HDAC8

**Figure 13:**
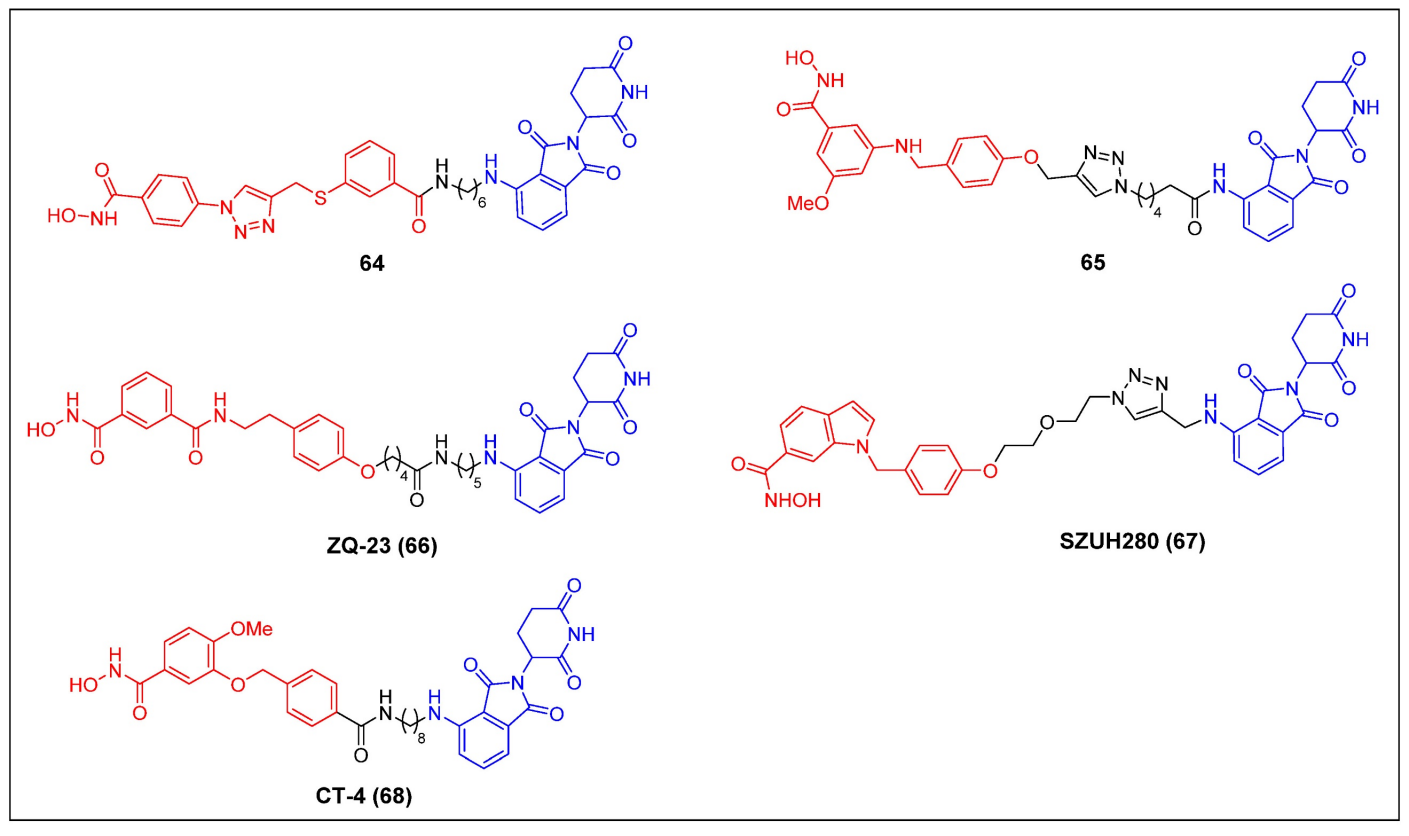
HDAC8 PROTACs (red for E3 ligand, blue for POI ligand and black for linker).

Since 2022, five CRBN-recruiting HDAC8 PROTACs have been reported (Figure 12). The first reported HDAC8 PROTAC, compound 64 (Figure 13), was designed based on an HDAC8-selective inhibitor 68. It exhibited potent and selective HDAC8-degrading activity (DC_50_ = 702 nM) without affecting the levels of HDAC1, HDAC2, and HDAC6 in Jurkat cells. Notably, it suppressed the growth of Jurkat cells (GI_50_ = 0.78 μM) more potently than its parent compound (GI_50_ = 7.09 μM). Darwish *et al*. showed several HDAC8 PROTACs depended on a potent and selective benzhydroxamic HDAC8 inhibitor 69. Among these PROTACs, compound 65 (Figure 13) exhibited the highest potency, with an IC_50_ value of 0.25 µM against HDAC8. Notably, it displayed favorable selectivity towards HDAC6 (IC_50_ = 17.2 µM) and HDAC1 (IC_50_ = 16.2 µM). Additionally, compound 65 had a significant impact on the formation of colonies, while demonstrating limited cytotoxicity towards HEK293 cells. The HDAC8 level in an SK-N-BE (2)-C cell lysate, treated with compound 65 at a concentration of 10 µM for a duration of 10 hours, exhibited a reduction to 30%. The administration of compound 65 has induced a notable increase in the acetylation levels of SMC3, a protein involved in the regulation of chromosome cohesion. Furthermore, the application of compound 65 to neuroblastoma cells has led to the process of neuronal differentiation, indicating its potential therapeutic value in promoting neural development. Chen *et al*. developed a collection of HDAC8 degraders through the conjugation of a dual HDAC6/8 inhibitor BRD73954 with pomalidomide 70. Among the group of HDAC8 degraders, ZQ-23 (compound 66) (Figure 13) demonstrated significant efficacy and specificity in degrading HDAC8. In HCT-116 cells at a concentration of 5 µM, ZQ-23 exhibited a DC_50_ value of 147 nM and achieved a D_max_ of 93% for HDAC8. Importantly, ZQ-23 did not exhibit any discernible effects on other HDAC isoenzymes, including HDAC1 and HDAC3. Using a different POI ligand, Zhu *et al*. created SZUH280 (compound 67) (Figure 13) by linking the HDAC8 inhibitor PCI-34051 to pomalidomide using a PEG linker 71. It was able to effectively cause HDAC8 degradation in A549 cells (DC_50_ = 580 nM) and inhibit A549 cell growth at micromolar concentrations. SZUH280 hampered DNA damage repair in cancer cells, enhancing cellular radiosensitization. The administration of SZUH280 in nude mice harboring A549 cells resulted in the fast and sustained degradation of HDAC8 protein. Furthermore, the administration of SZUH280 alone or in conjunction with irradiation led to sustained tumor regression in a murine model with A549 tumors. More recently, Zhao *et al*. also created a potent and selective HDAC8 PROTAC, CT-4 (compound 68) (Figure 13), by linking an HDAC8 inhibitor to pomalidomide through an aliphatic linker 72. The results demonstrated a DC_50_ value of 1.8 nM and a D_max_ value of 97% in MDA-MB-231 cells. Similarly, a DC_50_ value of 4.7 nM and a D_max_ value of 95% were observed in Jurkat cells. Significantly, CT-4 exhibited substantial inhibitory effects on cell migration while displaying limited effects on cell proliferation in MDA-MB-231 cells. The Jurkat cell line exhibited significant anti-proliferative effects upon treatment with CT-4, primarily mediated through the activation of apoptosis.

#### 3.1.3 SIRT2

**Figure 14:**
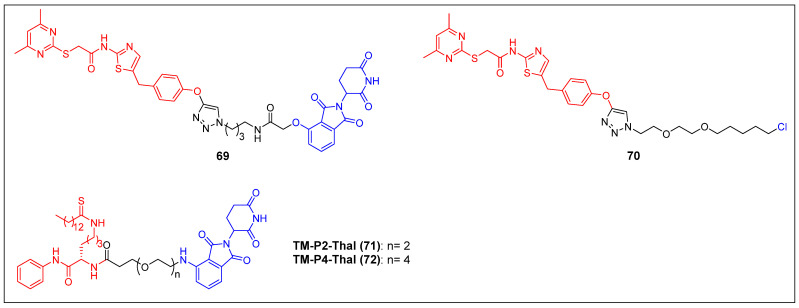
SIRT2 PROTACs (red for E3 ligand, blue for POI ligand and black for linker).

The initial discovery of a Sirt2 PROTAC (compound 69) (Figure 14), as documented by Schiedel *et al*., involved the fusion of a highly effective and isotype-specific Sirt2 inhibitor with thalidomide 73. In HeLa cells, PROTAC 58 selectively and dose-dependently induced Sirt2 degradation (IC_50_ = 0.25 µM). The group then developed a chloroalkylated Sirt2 PROTAC (compound 70) (Figure 14) that recruits the Parkin E3 ligase to degrade Sirt2 at a 10-fold smaller concentration than the CRBN-mediated PROTAC 69 74. Subsequently, Lin *et al*. reported two new SIRT2 degraders—TM-P2-Thal (compound 71) and TM-P4-Thal (compound 72) (Figure 14)—formed by conjugating the selective SIRT2 inhibitor TM to the CRBN ligand using PEG linkers 75. These two PROTACs can effectively degrade SIRT2 in breast cancer cells and exhibit antiproliferative effects in other cancer cells as well.

#### 3.1.4 Other HDACs

**Figure 15:**
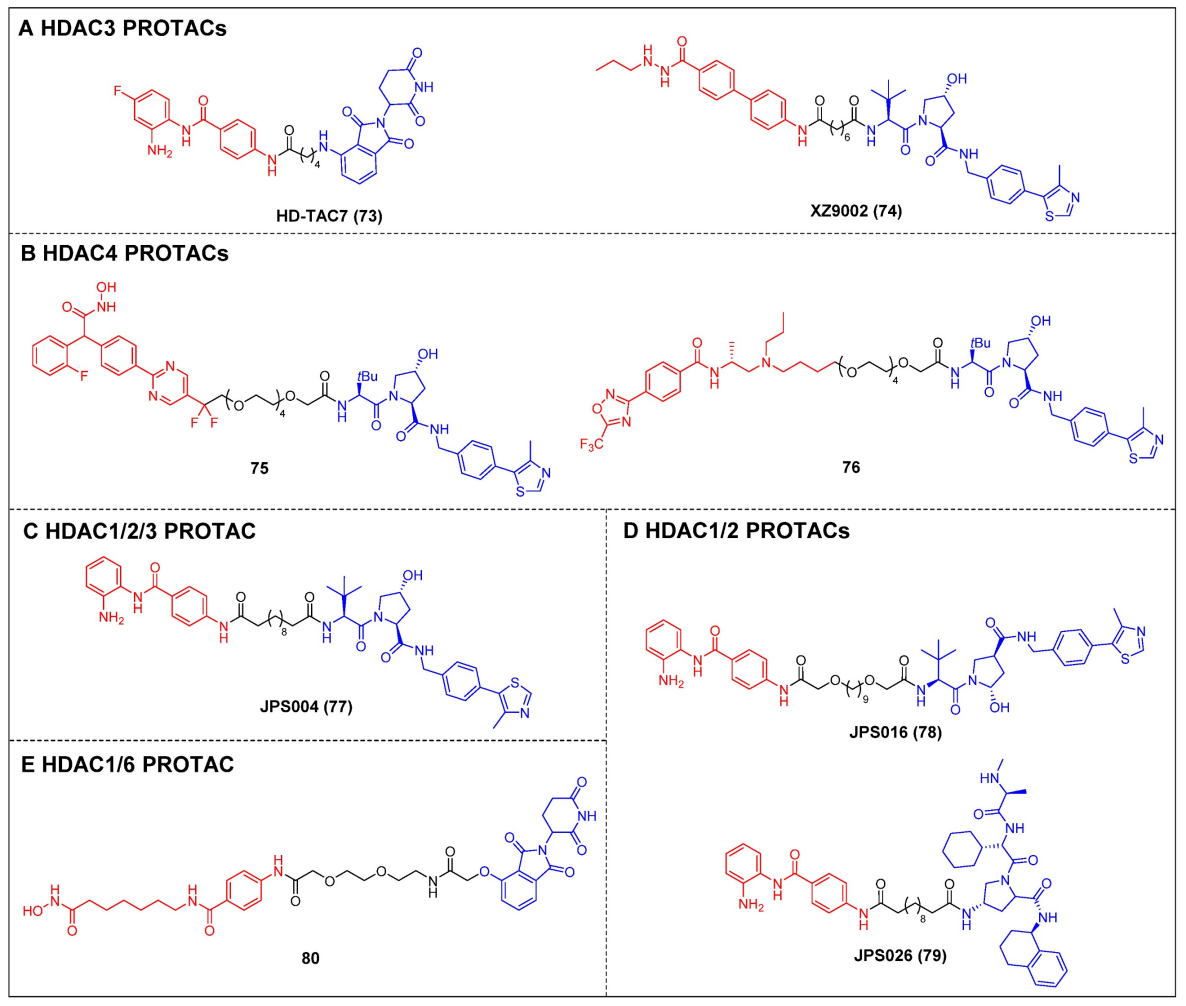
Other HDACs PROTACs (red for E3 ligand, blue for POI ligand and black for linker). HDAC3 (A), HDAC4 (B), HDAC1/2/3 (C), HDAC1/2 (D), HDAC4 (E).

Dekker *et al*. created a group of selective HDAC3 PROTACs through the conjugation of the o-amino anilide-based class I HDAC inhibitor C1994 with the CRBN ligand pomalidomide 148. Among the analogs tested, HD-TAC7 (compound 73) (Figure 15A) exhibited the highest activity against HDAC3, with an IC_50_ value of 1.1 nM. In RAW264.7 macrophages, HD-TAC7 demonstrated selective and potent induction of HDAC3 degradation (DC_50_ = 0.32 μM) without affecting HDAC1 and 2. The same year, Xiao *et al*. developed a VHL-recruiting HDAC3 PROTAC, XZ9002 (compound 74) (Figure 15A), based on their previously documented class I HDAC inhibitor SR-3558 149. In MDA-MB-468 cells, XZ9002 selectively dose- and time-dependently induced HDAC3 degradation (DC_50_ = 42 nM) with negligible alterations in HDAC1, 2, or 6 levels. Furthermore, XZ9002 exhibited substantial antiproliferative activity in the breast cancer cell lines T47D, BT549, and HCC1143.

The initial discovery of highly effective and specific HDAC4 PROTACs was documented by Macabuag *et al* 150. Compounds 75 and 76 (Figure 15B) emerged as the most potent VHL-based degraders, originating from two distinct series of pan class IIa HDAC inhibitors. These compounds exhibited significant abilities for degrading HDAC4 in primary neurons. Notably, their potent activity—potentially linked to slow nascent HDAC4 protein synthesis in these neurons—resulted in a concentration-dependent reduction of HDAC4 levels within the low nanomolar range.

The development of VHL-based PROTAC molecules was reported by Hodgkinson *et al*. that targeted class I HDAC (HDAC1/2/3) by using a class I HDAC-selective inhibitor CI-994 151. Among these molecules, the most active degrader, JPS004 (compound 77) (Figure 15C), demonstrated a DC_50_ of 1 µM for HDAC 1/2 and effectively reduced the viability of colon cancer HCT116 cells. Following this, by investigating the impacts of linker length and composition based on JPS004, Smalley *et al*. identified a more potent degrader, JSP016 (compound 78) (Figure 15D; HDAC1, DC_50_ = 550 nM; HDAC2, DC_50_ = 530 nM) 152. They also developed a novel PROTAC, JPS026 (compound 79) (Figure 15D), which used IAP as an E3 ligase ligand 153. JPS026 not only induced the degradation of HDAC1/2 but also triggered apoptosis and suppressed DNA replication, exhibiting a higher efficacy for inducing cell death compared to JSP004.

Hansen *et al*. reported the solid-phase synthesis of an HDAC degrader 80 (Figure 15E), which was formed by conjugating SAHA and thalidomide using a PEG linker 154. Compound 80 was found to degrade HDAC6 and HDAC1, as well as induce the hyperacetylation of histone H3 and α-tubulin in the AML cell line HL60.

### 3.2 ENL

The YEATS domain, which derives its name from its constituent members Yaf9, eleven-nineteen-leukemia (ENL), ALL1-fused gene from chromosome 9 (AF9), transcription initiation factor TFIID subunit 14 (Taf14), and Sas5, is a type of histone acetylation “reader” that has been evolutionarily conserved from yeasts to humans 155. One of the proteins that have an essential function in AML development is the ENL protein 156. The induction of an anti-leukemia effect and inhibition of leukemia growth can be achieved through the ENL depletion or interruption of the interaction between its YEATS domain and acetylated histones. These findings revealed that targeting the YEATS domain of ENL holds promise as a therapeutic strategy.

**Figure 16:**
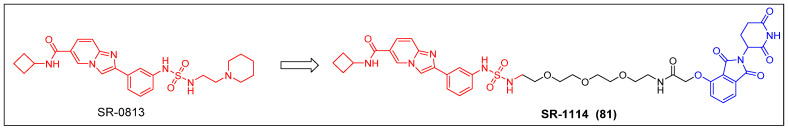
ENL PROTAC SR-1114 (red for E3 ligand, blue for POI ligand and black for linker).

The process of discovery chemistry was initiated with a well-coordinated endeavor involving high-throughput chemical screening; Erb *et al*. discovered a potent and selective amido-imidazopyridine-based ENL YEATS domain inhibitor, SR-0813 (*K*_d_ = 30 nM, IC_50_ = 25 nM) (Figure 16) 157. Based on the findings of SR-0813, the researchers proceeded to design and synthesize a first-in-class degrader SR-1114 (compound 81) (Figure 16) that targeted ENL. SR-1114 was able to cause ENL degradation in various cell lines and showed its highest activity in MV4-11 cells, with a DC_50_ of 150 nM. This study offers the initial pharmacological confirmation of the ENL YEATS domain as a viable target in mixed lineage leukemia (MLL)-fusion leukemia. Additionally, a meticulously characterized chemical probe was developed to facilitate the investigation of ENL/AF9 YEATS domains.

## 4. Histone methylation-/demethylation-related targets

### 4.1 PRC

As members of the histone methyltransferase family, the polycomb group (PcG) of proteins, including polycomb repressive complex 1 (PRC1) and 2 (PRC2), can regulate gene transcription 158. PRC1 functions in a hierarchical manner in which, within the catalytic core, really interesting new gene 1A/B (RING1A/B) selectively interacts with one of the six PcG RING fingers 1-6 (PCGF1-6) paralogs, which in turn controls the mono-ubiquitination of histone H2A at Lys119 (H2AK119ub) 159. B-cell specific Moloney murine leukemia virus integration site 1 (BMI1) and RING1B are integral components of the primary heterodimeric complex of canonical PRC1 and are known to have significant implications in the advancement of various types of cancer 160. PRC2 is a multicomponent conserved transcriptional repressive complex that functions by facilitating the addition of mono-, di- and trimethyl groups to lysine residue 27 on histone H3 (known as H3K27me1/2/3) 161. The fundamental constituents of the PRC2 complex comprise enhancer of the zeste homolog 1/2 (EZH1/2), suppressor of the zeste 12 protein homolog (SUZ12), embryonic ectoderm development (EED), and retinoblastoma (Rb)-associated proteins 46/48 (RbAp46, or RBBP7/RbAp48, or RBBP4) 162. PRC2 components have been reported to be overexpressed in multiple cancer types and have been associated with cancer progression 163. However, the availability of therapeutic agents that selectively target PRC1/2 is currently limited.

**Figure 17:**
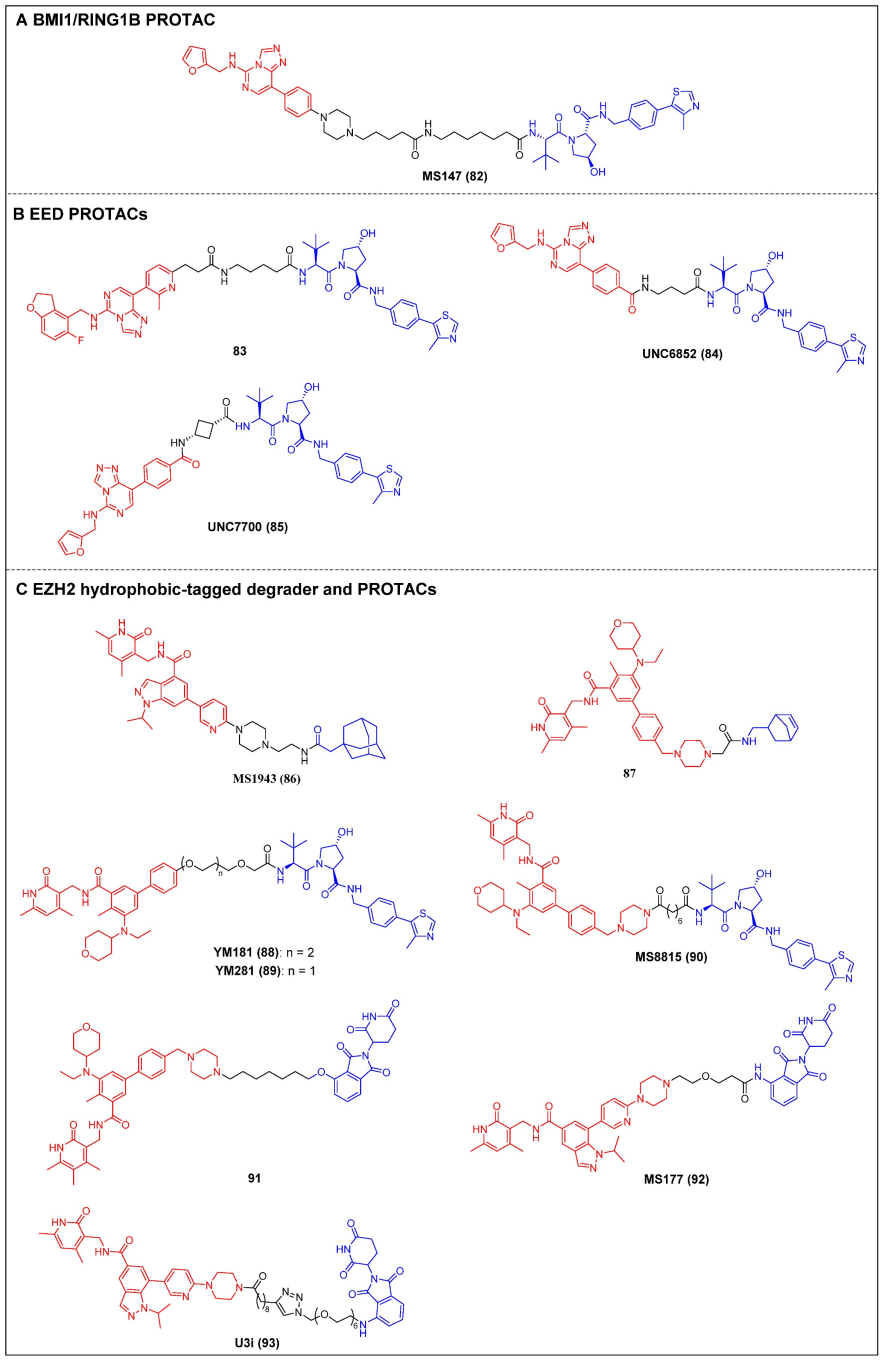
PRC degraders (red for E3 ligand, blue for POI ligand and black for linker). (A) BMI1/RING1B PROTAC; (B) EED PROTAC; (C) EZH2 hydrophobic-tagged degrader and PROTAC.

Recently, Jin *et al*. reported the first PRC1 degrader, MS147 (compound 82) (Figure 17A), which was composed of an EED binder (EED226) and a VHL ligand 164. MS147 exhibited a higher propensity for the degradation of BMI1 and RING1B compared to other PRC2 components EED, EZH2, and SUZ12. In addition, it successfully decreased the levels of H2AK119ub, a process catalyzed by PRC1, while leaving the levels of PRC2-mediated H3K27me3 unchanged. Moreover, MS147 demonstrated inhibition of cell proliferation in various cancer cells that exhibit resistance to EZH2 knockout (KO) or EED/PRC2 degraders.

The first EED-targeting PROTAC, compound 83 (Figure 17B), was created by conjugating a EED inhibitor MAK683 to a VHL ligand 76. Compound 83 potently inhibited the enzymatic activity of PRC2 (pIC_50_ = 8.11). It selectively degraded EED (D_max_ = 95%), EZH2 (D_max_ = 95%), and SUZ12 (D_max_ = 80%), and it was observed that the proliferation of both the EZH2 mutant diffuse large B-cell lymphoma (DLBCL) cell line and the EZH2 wild-type (WT) rhabdoid cancer cell line was effectively suppressed. In the same year, James *et al*. published another EED-targeting PROTAC named UNC6852 (compound 84) (Figure 17B), which was based on a PRC2 allosteric inhibitor (EED226) and a VHL ligand 77. In HeLa cells, UNC6852 selectively degraded EED (DC_50_ = 0.79 µM) and EZH2 (DC_50_ = 0.3 µM). It can also reduce the levels of H3K27me3 and potently inhibit the proliferation of diffuse large B-cell lymphoma (DB) and Pfeiffer cells (DLBCL-related cell lines bearing mutated EZH2). Recently, they further reported a novel potent EED-targeting PRC2 degrader, UNC7700 (compound 85) (Figure 17B), which features a rigidity *cis*-cyclobutane linker in contrast to the flexible propyl linker of UNC6852 78. UNC7700 exhibited a strong degradation activity against EED (DC_50_ = 111 nM; D_max_ = 84%) and EZH2^WT^/EZH2^Y641N^ (DC_50_ = 275 nM; D_max_ = 86%) in a diffuse large B-cell lymphoma DB cell line. It also demonstrated promising anti-proliferative effects in DB cells (EC_50_ = 0.79 μM).

Jin *et al*. synthesized a first-in-class EZH2-selective degrader, MS1943 (compound 86) (Figure 17C), by linking an EZH2 inhibitor (C24) to a hydrophobic adamantyl group 79. MS1943 degraded EZH2 in MDA-MB-468 cells at 5 µM effectively, suppressed H3K27me3 significantly, and induced cell death in EZH2-dependent TNBC cells, but not in normal cells. Xu *et al*. discovered norbornene as a novel Hyt and designed the tazemetostat-based EZH2 degrader 87 (Figure 17C) 80. Degrader 87 significantly decreased EZH2 levels at 10 μM and achieved complete degradation at 40 μM. Furthermore, it demonstrated a remarkable enhancement in the antiproliferative activity of MDA-MB-468 cells compared to tazemetostat.

Two VHL-recruiting tazemetostat-based EZH2 PROTACs were reported by two research groups in 2021. Wen *et al*. created the initial class of EZH2 degraders, of which the two most potent members, YM181 (compound 88) and YM281 (compound 89) (Figure 17C), exhibited effective antiproliferative activities in DLBCL, as well as in other subtypes of lymphoma cell lines 81. Furthermore, YM181 and YM281 exhibited superior antiproliferative effects in lymphoma xenografts and patient-derived primary lymphoma cells without obvious toxicities at their effective doses. In the same year, Jin *et al*. published a study describing an additional novel, highly effective, and specific PROTAC molecule, MS8815 (compound 90) (Figure 17C), that recruits VHL to degrade EZH2. This was synthesized by substituting the morpholinyl group of EPZ643836 with a piperazine group and connecting it to a VHL ligand 82. Notably, MS8815 induced a significant degradation of EZH2 in the TNBC cell lines MDA-MB-453 and BT549, with a DC_50_ of 140 nM for a 48-h treatment. MS8815 exhibited a slightly higher efficacy compared to YM281 in terms of degrading EZH2 and suppressing proliferation in various TNBC cell lines. Conversely, tazemetostat did not demonstrate any impact on EZH2 degradation or cell growth inhibition. In addition, it was observed that MS8815 exhibited bioavailability in mice, as evidenced by the attainment of adequate plasma exposure levels following intraperitoneal (IP) administration. Yu *et al*. also developed CRBN-based EZH2 PROTACs derived from tazemetostat 83. Among them, compound 91 (Figure 17C) exerted the most potent inhibitory effect on EZH2 (IC_50_ = 2.7 nM), surpassing that of tazemetostat (IC_50_ = 3.7 nM). It also induced degradation of the PRC subunits EZH2, SUZ12, EED, and RbAp in WSU-DLCL-2 cells 165. Additionally, compound 91 exhibited significant antiproliferative activities against SWI/SNF-mutant cancer cells that are dependent on the enzymatic and nonenzymatic activities of EZH2. Wang *et al*. developed an efficient CRBN-targeting EZH2 degrader, MS177 (compound 92) (Figure 17C), depending on the selective EZH2 inhibitor C24 (Figure 17C), which can target EZH2, SUZ12, and EED 84. MS177 downregulated the level of H3K27me3 and effectively induced c-Myc degradation in MM cells. Moreover, it could reactivate immune response genes and effectively suppress the proliferation of MM cells 166. The same year, Wang and Kong reported a novel potent EZH2 PROTAC, U3i (compound 93) (Figure 17C), bearing a triazole linker also based on C24 and the CRBN ligand, which exhibited a strong affinity for PRC2 *(K*_D_ = 16.19 nM) 167. Notably, U3i showed potent inhibitory activity against MDA-MB-231 (IC_50_ = 0.57 μM) and MDA-MB-468 cells (IC_50_ = 0.38 μM). It successfully triggered the degradation of EZH2, SUZ12, and EED in two TNBC cell lines, resulting in apoptotic cell death while showing minimal cytotoxicity toward normal cells.

### 4.2 WDR5

The chromatin-associated WD40 repeat domain protein 5 (WDR5) is associated with chromatin functions as a subunit of the MLL histone methyltransferase complexes. These complexes are responsible for the enzymatic process of methylating H3K49 168. The association of WDR5 with the initiating and developing of various cancers has been reported, but also with poor clinical prognoses, making it an appealing drug target 169. Despite the development of selective WDR5 inhibitors, their limited antiproliferative effects and poor *in vivo* activity have spurred the exploration of alternative therapeutic options 86, 170. The utilization of chemical means to induce proteasomal degradation of WDR5 may, therefore, serve as a sophisticated strategy for effectively targeting all oncogenic functions.

**Figure 18:**
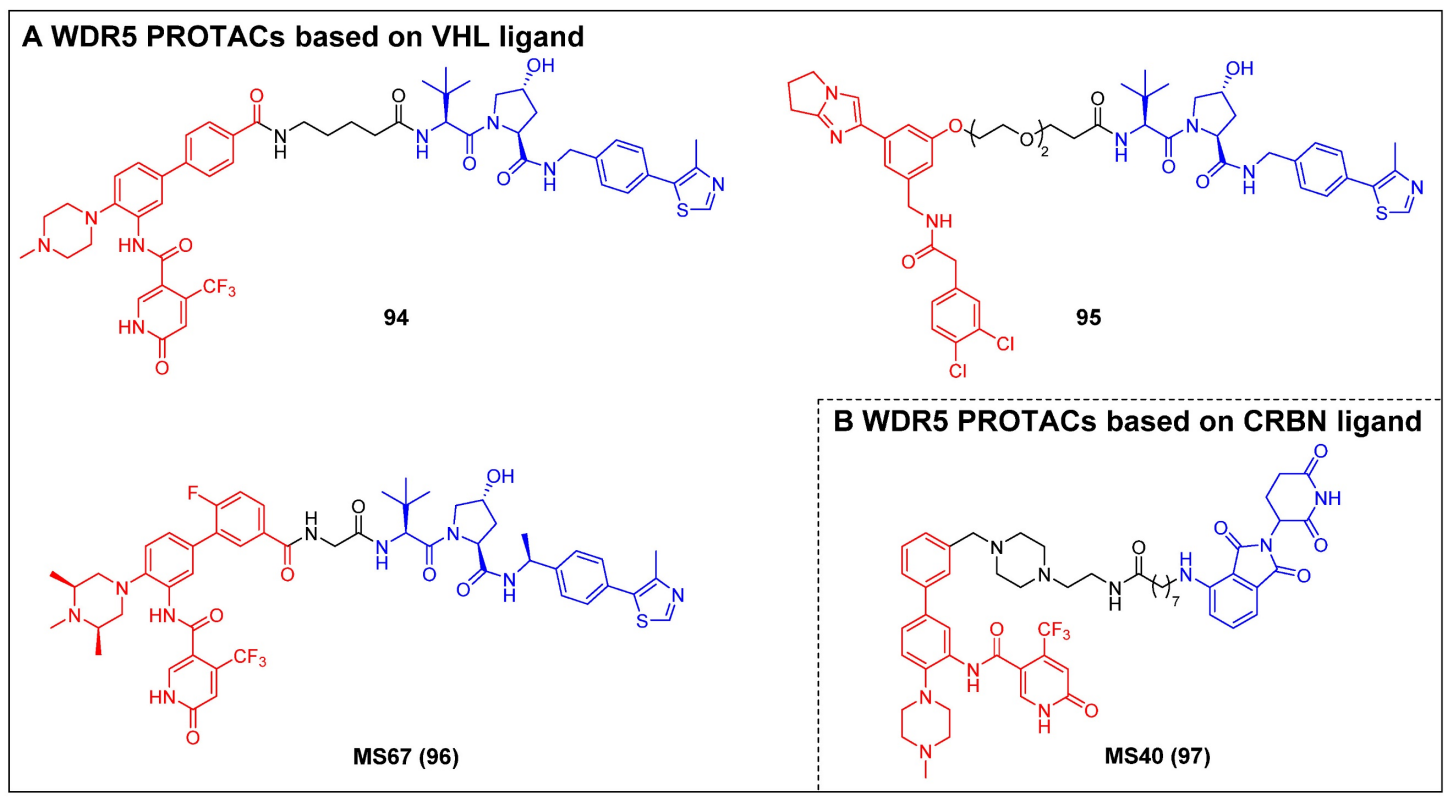
WDR5 PROTACs (red for E3 ligand, blue for POI ligand and black for linker) based on VHL ligand (A) and CRBN ligand (B).

Knapp *et al*. reported two types of VHL-based WDR5 degraders depended on various WIN site binding scaffolds: the (trifluoromethyl)-pyridine-2-one-based OICR-9429 group and the pyrroloimidazole-based WDR5 antagonists 85. In MV4-11 cells, the OICR-9429-derived degrader 94, with a four-carbon linker (Figure 18A; DC_50_ = 53 nM, D_max_ = 58%), and the pyrroloimidazole-based degrader 95 (Figure 18A; DC_50_ = 1.24 µM, D_max_ = 53%) with two PEG linkers were the two most potent ones. MS67 (compound 96) (Figure 18A), a WDR5 degrader composed of a phenylbenzamide-scaffolded WDR5 binder and a VHL ligand, was designed by Jin *et al*. in 2021. MS67 selectively degraded WDR5 (DC_50_ = 3.7 nM) and effectively suppressed the growth of MLL-rAML cells and pancreatic ductal adenocarcinoma (PDAC) cells *in vitro* 86. It also significantly inhibited tumor growth in an AML animal model and a PDX model. The same group later reported the development of a novel WDR5 PROTAC, MS40 (compound 97) (Figure 18B), through the connection between WDR5 binder phenylbenzamide and lenalidomide, utilizing a linker composed of a piperazine-containing alkylamide 87. Notably, MS40 could induce the simultaneous degradation of WDR5, CRBN, and Ikaros zinc finger (IKZF) transcription factor members such as IKZF1 and IKZF3, which are recognized neo-substrates of immunomodulatory drugs (IMiDs). The degradation of WDR5 by MS40 led to the dissociation of the MLL/KMT2A complex from chromatin, thereby causing a decrease in the levels of H3K4me2. MS40 was also found to effectively suppress the transcription of target genes associated with both WDR5 and IKZF, long with inhibiting the growth of primary leukemia cells.

### 4.3 NSD

Lysine methyltransferases, also known as HKMTases, are a class of epigenetic regulators responsible for facilitating the transfer of 1-3 methyl groups onto specific lysine residues (K3, K9, K20, K27, K36, and K79) located on histones H3 and H4 171. The nuclear receptor-binding Su (var)3-9, Enhancer-of-zeste and Trithorax (SET) domain (NSD) family, a group of HKMTases, is devided into three subtypes: NSD1, NSD2 (MMSET/WHSC1), and NSD3 (WHSC1L1). The NSDs are essential enzymes involved in the methylation of histone H3 lysine 36 (H3K36), which specifically catalyze the mono- and di-methylation modifications of this histone residue 172. Overexpression, mutations, and translocations of NSDs are linked with many of human malignancies 173. Hence, all three entities are regarded as significant subjects for the advancement of innovative pharmaceuticals aimed at combating cancer.

**Figure 19:**
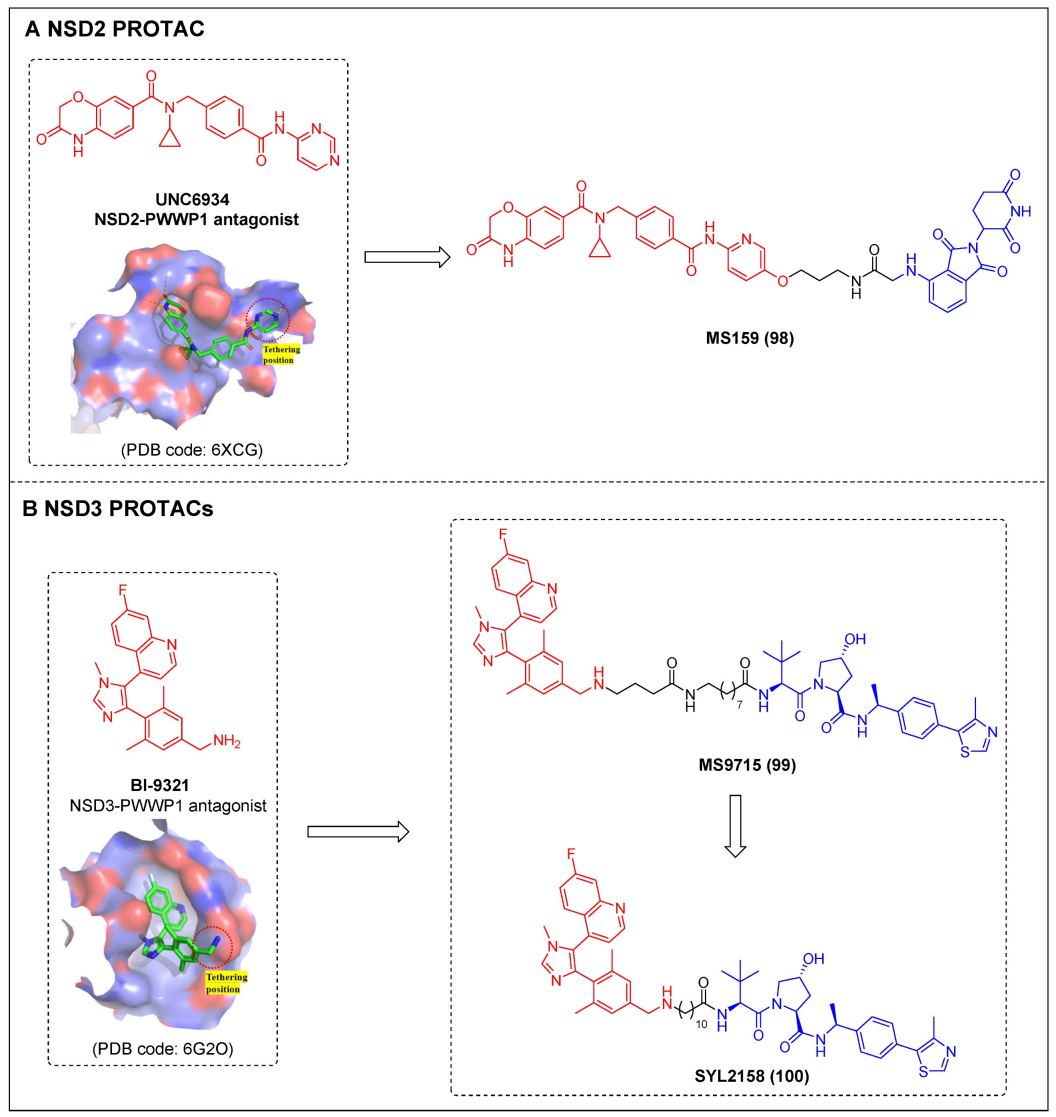
NSD PROTACs (red for E3 ligand, blue for POI ligand and black for linker). NSD2 (A) and NSD3 (B) PROTACs.

Recently, the first-in-class NSD2 PROTAC MS159 (compound 98) (Figure 19A) was reported by Jin *et al* that combined the selective NSD2-PWWP1 antagonist UNC6934 with a CRBN E3 ligase ligand 88. MS159 demonstrated efficient degradation of NSD2 in 293FT cells through a mechanism that is dependent on concentration, time, CRBN, and UPS. In addition, it demonstrated effective degradation of the CRBN neo-substrates IKZF1 and IKZF3 in two MM cell lines while not affecting GSPT1. It is worth mentioning that MS159 exhibited a greater ability to inhibit cell growth in KMS11 and H929 MM cells compared to its precursor NSD2-PWWP1 antagonist UNC6934. This finding suggests that the potential therapeutic efficacy of pharmacologically degrading NSD2 and IKZF1/3 may surpass that of pharmacologically antagonizing NSD2-PWWP1 and chromatin protein-protein interactions (PPIs).

Xu *et al*. synthesized NSD3-targeting PROTACs based on the NSD3-PWWP1 antagonist BI-9321 89. Among these compounds, a VHL-recruiting PROTAC, MS9715 (compound 99) (Figure 19B), showed the highest NSD3 degradation activity, with a DC_50_ of 4.9 µM and a D_max_ exceeding 80% in MOLM-13 cells. Global proteomic profiling experiments later confirmed that MS9715 was highly selective for NSD3 and that its effects resembled those observed in NSD3 KO studies. In addition, MS9715, but not BI-9321, depleted cellular NSD3 and suppressed c-Myc-associated genes in NSD3-dependent hematological cancer cells. Sun *et al*. also used BI-9321 to design a class of NSD3 PROTACs 90. The implementation of significant linker modifications resulted in the development of the highly effective and specific NSD3 degrader, SYL2158 (compound 100) (Figure 19B). This compound demonstrated successful induction of NSD3 degradation in lung cancer cell lines NCI-H1703 (DC_50_ =1.43 μM) and A549 (DC_50_ =0.94 μM), respectively. The compound exhibited preferential activity towards NSD1 and NSD2, resulting in a decrease in methylation of H3K36. Additionally, SYL2158 effectively decreased the NSD3 protein level in A549 xenograft mouse models.

### 4.4 Other histone methylation-related targets

The process of arginine methylation is a prevalent epigenetic modification observed in nuclear and cytoplasmic proteins. This modification is facilitated by enzymes known as protein arginine methyltransferases (PRMTs) 174. Protein arginine methyltransferase 5 (PRMT5), a type II PRMT, is responsible for facilitating the symmetrical dimethylation process at arginine residues on histone or non-histone substrates, thus participating in a number of biological processes 175. Although multiple PRMT5 inhibitors have been reported, none are capable of eliminating the scaffolding functions of PRMT5 176, 177. Therefore, the degraders of PRMT5 presents a potentially advantageous therapeutic strategy for the treatment of diverse medical conditions.

**Figure 20:**
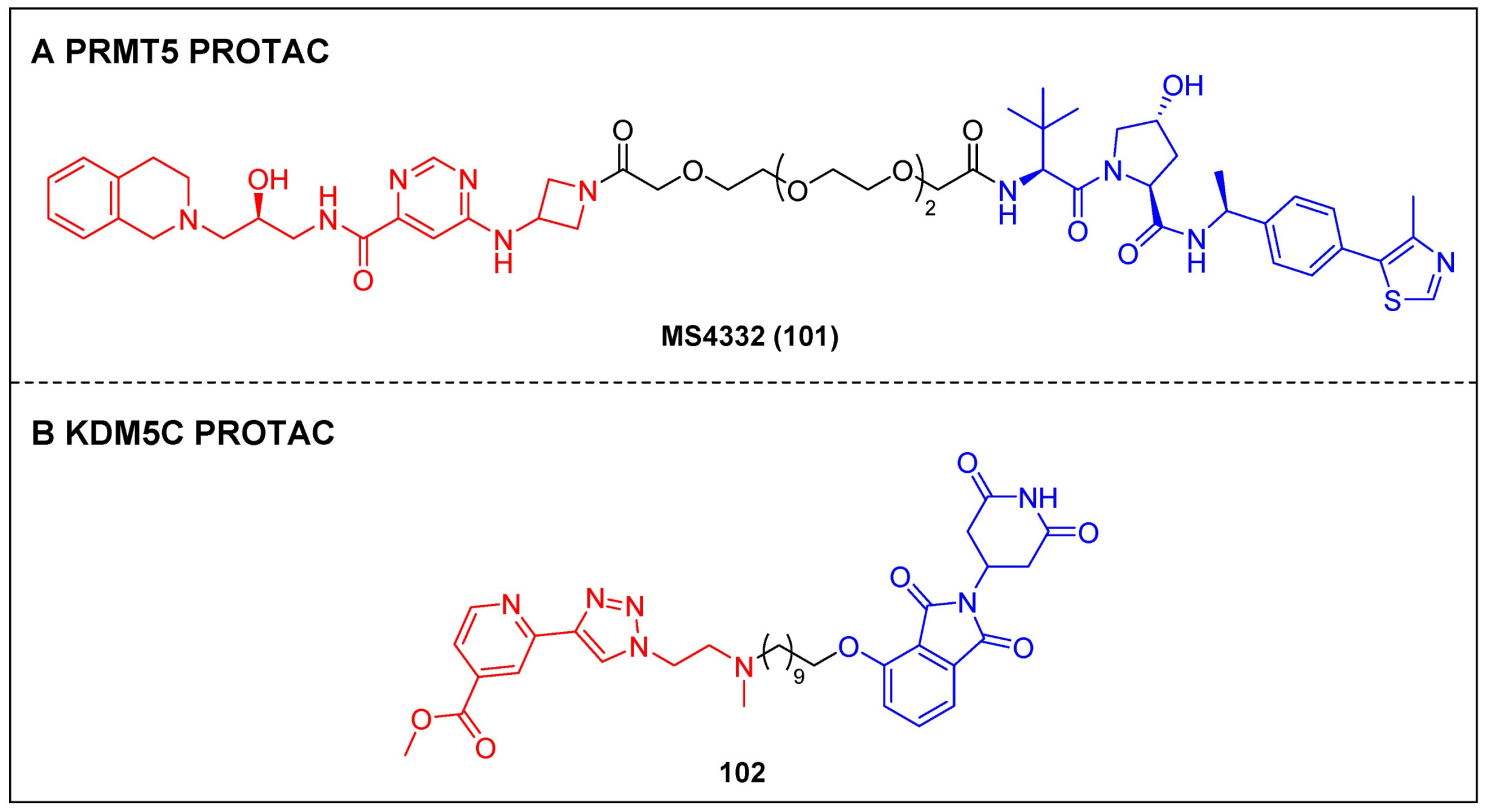
PRMT5 (A) and KDM5C (B) PROTACs (red for E3 ligand, blue for POI ligand and black for linker).

Shen *et al*. developed the first PRMT5 degrader, MS4322 (compound 101) (Figure 20A), by conjugating the existing PRMT5 inhibitor EPZ015666 to a VHL ligand (S, R, S)-AHPC-Me (VHL-2) using a PEG linker 178. MS4322 demonstrated significant efficacy for reducing PRMT5 levels in MCF-7 breast cancer cells (DC_50_ = 1.1 µM, D_max_ = 74%) and suppressed cell growth in multiple cancer cell lines such as HeLa, A549, A172, and Jurkat. It also exhibited a highly selective inhibition of PRMT5, as evidenced by a global proteomics analysis conducted in MCF-7 cells. Notably, MS4322 demonstrated favorable plasma exposure in a mouse PK study.

Lysine demethylase 5C (KDM5C), alternatively referred to as JARID1C or SMCX, serves as a demethylase specifically targeting H3K4me2/3 in mammalian cells 179. KDM5C is widely acknowledged as a tumor suppressor due to its role in modulating enhancer activities across multiple cancer types, including prostate, breast, cervix, and clear-cell renal carcinoma 180. Despite some RNA interference (RNAi) experiments, which indicated that KDM5C defects exhibited anticancer effects, the efficacies of several reported KDM5C inhibitors for exerting potent anticancer effects remain limited 181. Hence, the development of degraders that specifically target KDM5C to inhibit both its catalytic and scaffolding functions may represent a viable solution.

Suzuki *et al*. reported the first histone demethylase degrader, compound 102 (Figure 20B), by conjugating their previously identified KDM5C inhibitor with thalidomide as an E3 recruiter 182. Compound 102 exhibited remarkable degradation activity against KDM5C and superior inhibitory impacts on the growth of prostate cancer PC-3 cells compared to its parent inhibitor. Further cellular studies revealed that compound 102 could elevate the levels of both H3K4me3 and H3K27Ac, which was induced by the direct inhibition of the catalytic function of KDM5C, as well as the indirect inhibition of HDAC through the suppression of the KDM5C's scaffolding function.

## 5. Post-translational modification-related targets

### 5.1 EGFR

EGFR, also known as ErbB1/HER1, contains an intracellular kinase region, transmembrane region and extracellular ligand-binding region 183. The overexpression and aberrant expression of EGFR have been observed in numerous solid tumors, exerting control over cell proliferation, migration, and survival 184. A number of tyrosine kinase inhibitors (TKIs) and monoclonal antibodies (mAbs) and antibody drug conjugates (ADCs) have been approved for targeting the intracellular and extracellular domains of EGFR 183. Nevertheless, the practical utility of EGFR is constrained by the presence of EGFR mutations, cancer heterogeneity, and the inevitable development of drug resistance. To overcome these limitations, protein degraders have been gaining momentum for their potential as a promising anti-EGFR therapy.

**Figure 21:**
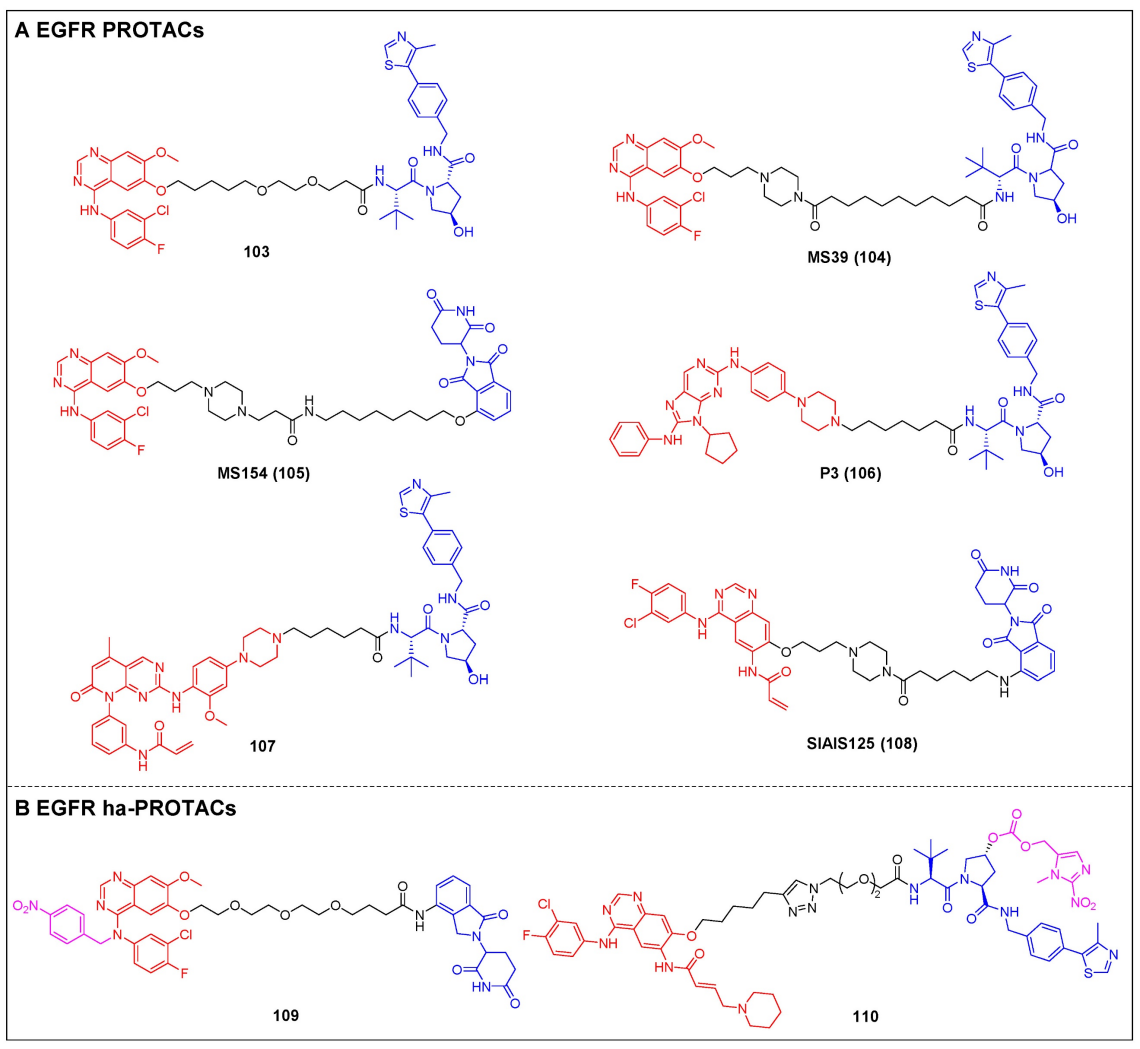
EGFR degraders (red for E3 ligand, blue for POI ligand, black for linker, and purple for HALG).

In 2018, Crews group pioneered the development of the first series of EGFR PROTACs by conjugating the various EGFR inhibitors (lapatinib, gefitinib, and afatinib) with the VHL ligand 91. Among these, compound 103 (Figure 21A), utilizing gefitinib as the warhead, effectively degraded the EGFR^del19^ (DC_50_ = 11.7 nM) in the HCC827 cell line and EGFR^L858R^ (DC_50_ = 22.3 nM) in the H3255 cell line. It is noteworthy that the degradation of EGFR^L858R^ induced by compound 103 demonstrated significant suppression of programmed death receptor ligand 1 (PD-L1) and indoleamine-2,3-dioxygenase-1 (IDO1) levels in H3255 cells, which enhance anti-tumor immune response in NSCLC 92. Jin *et al* reported a newly discovered gefitinib-based VHL-recruiting EGFR degrader MS39 (compound 104) (Figure 21A) and a first CRBN-based EGFR degrader MS154 (compound 105) (Figure 21A) 93. MS39 (HCC827 cells: DC_50_ = 5 nM; H3255 cells: DC_50_ = 3.3 nM) and MS154 (HCC827 cells: DC_50_ = 11 nM; H3255 cells: DC_50_ = 25 nM) effectively induced the degradation of mutant EGFR in cancer cells. Zhang group developed several EGFR PROTACs by integrating a reversible EGFR-TKI bearing purine ring with CRBN or VHL ligand 94. Notably, VHL-based PROTAC P3 (compound 106) (Figure 21A) demonstrated significant efficacy in inducing the degradation of mutant EGFR in HCC-827 (EGFR^del19^) (DC_50_ = 0.51 nM) and H1975 cells (EGFR^L858R/T790M^) (DC_50_ = 126.2 nM), respectively. MS154 also showed potent anti-proliferative activity against HCC-827 (IC_50_ = 0.83 nM) and H1975 cells (IC_50_ = 203.01 nM). Ding group reported EGFR^L858R/T790M^ PROTACs based on a selective EGFRL^858R/T790M^ inhibitor XTF-262 95. The most potent degrader 107 (Figure 21A), incorporating a VHL ligand, induced EGFR^L858R/T790M^ (DC_50_ = 5.9 nM) degradation effectively and exhibited significant antiproliferative activity against H1975 cells (IC_50_ = 506 nM). Recently, Jiang group designed a novel class of highly selective and functional EGFR PROTACs based on an EGFR inhibitor canertinib and CRBN ligand pomalidomide, as exemplified by SIAIS125 (compound 108) (Figure 21A) 96. SIAIS125 effectively induced the degradation of EGFR^L858R/T790M^ in H1975 cells and EGFR^e19d^ in PC9 cell lines, but did not affect EGFR^Ee19d/T790M^ in PC9Brca1 cells or EGFR^WT^ in A549 lung cancer cells. Mechanistic investigations of this work provided the first evidence that PROTAC-mediated degradation acted through both the autophagy and lysosomal system.

Zhang group disclosed the discovery of a hypoxia-activated PROTACs (ha-PROTACs) by installing a hypoxia-activated leaving group (HALG) to the 4-NH position on gefitinib 97. The obtained ha-PROTAC compound 109 (Figure 21B) induced significantly higher degradation of EGFR^del19^ under hypoxia condition than under normoxia condition in HCC4006 cells. Inspired by this work, Zhu group incorporated a nitroimidazole group, which is responsive to NTR, into the VHL ligand to develop a novel NTR-responsive PROTAC compound 110 (Figure 21B) 98. In normoxia condition, compound 110 was unable to degrade EGFR in HCC-827 cells. However, under hypoxia conditions, it released an active hydroxyl-containing PROTAC that demonstrated significant EGFR degradation activity (DC_50_ = 36.51 nM) and remarkable anti-proliferative activity (IC_50_ = 4.0 nM).

Bertozzi group pioneered the concept of LYTAC through the utilization of two cell-surface lysosome-targeting receptors (LTRs) to create the first-generation and the second-generation LYTACs, which depended on the cation-independent mannose-6-phosphate receptor (CI-M6PR) and asialoglycoprotein receptor (ASGPR) 99. Both two LYTACs achieved degradation of various membrane proteins, including EGFR.

### 5.2 USP7

Ubiquitin-specific protease 7 (USP7), also referred to as herpes virus-associated ubiquitin-specific protease (HAUSP), is a extensively characterized and most studied deubiquitinating enzyme (DUB). USP7 stabilizes MDM2, which facilitates the proteasomal degradation of tumor suppressor p53 185. Aberrant USP7 expression associated with a variety of human malignancies by regulating the activity of cancer-promoting or cancer-suppressing proteins and often correlates with unfavarable prognosis and metastasis 186. Hence, USP7 has been recognized as a reasonable target for cancer therapy, and several USP7-selective inhibitors have been developed in recent years 187.

**Figure 22:**
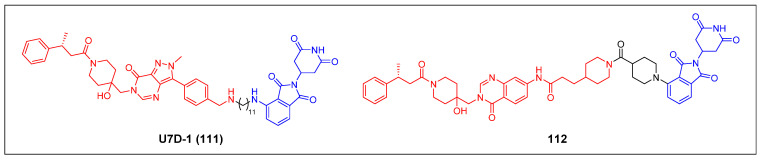
USP7 PROTACs (red for E3 ligand, blue for POI ligand and black for linker).

Zhou *et al*. reported the discovery of **U7D-1** (compound 111) (Figure 22), the first-generation small-molecule degrader, which efficiently induced USP7 degradation (DC_50_ = 33 nM) 100. **U7D-1** demonstrated significant inhibitory effects on the proliferation of both p53 wild-type and mutant cancer cells, while inhibitors showed no activity. Further mechanism study revealed that the regulation of the non-enzymatic functional regions (including apoptotic and E2F pathways) may contribute to the antiproliferative activities of USP7 degrader in p53 mutant cancer cells. Murgai *et al*. reported novel USP7 PROTACs based on a USP7 inhibitor **XL-188** 101. Among them, compound **112** (Figure 22) showed the most potent USP7 depletion with a D_max_ of 85% at 1 µM and a DC_50_ of 17 nM in MM cell line MM.1S. Furthermore, the rate of USP7 degradation was enhanced by increasing the concentration of compound **112**, eventually leading to a slight hook effect at 10 µM. In addition, **112** effectively induced apoptosis in several USP-dependent cancer cell lines.

## 6. Summary and perspective

The therapeutic potential of epigenetic therapies is being increasingly recognized, leading to the development of a diverse array of drugs that target epigenetic processes for the treatment of various diseases, including cancer. Notably, a number of these drugs have progressed to the stage of clinical trials 188. Since the discovery of the first PROTAC in 2001, TPD technologies have achieved remarkable progress in the degradation of epigenetic proteins, particularly “readers.” Compared to traditional small molecule inhibitors, PROTACs demonstrate enhanced target selectivity, heightened potency, prolonged duration of action, diminished side effects, elimination of scaffold function, and reduced likelihood of resistance. Consequently, these findings have the potential to provide additional therapeutic approaches for cancers that are dependent on epigenetic perturbations of gene expression. Moreover, these compounds could serve as valuable chemical instruments for investigating the functional significance of these epigenetic factors in biological processes 189. PROTACs, which target epigenetic proteins, recapitulate the post-translational effects of CRISPR-mediated genetic knockdown, thereby optimizing the targetability of these proteins. On the other hand, PROTACs have the potential to demonstrate significant enhancements in the selectivity of targeted degradation, surpassing the anticipated outcomes of their binary target engagement. Consequently, degraders can exhibit a higher degree of selectivity compared to inhibitors, particularly when targeting highly structural homologous proteins.

Despite the notable advancements in of PROTAC technology, several inherent limitations, including inadequate drug-like properties, uncontrolled catalytic activity leading to off-target effects, and restricted availability of E3 ubiquitin ligases, pose challenges that may hinder future progress 190. A pressing need arises for there is a more profound comprehension of the specific mechanisms governing PROTACs, both through experimentally exploration and computationally analysis 191. To monitor PROTAC-mediated degradation of BET proteins, researchers have introduced a novel and adaptable live-cell platform that leverages endogenous tagging technologies 192. Ciulli *et al*. developed a surface plasmon resonance (SPR)-based assayfor the quantification of PROTAC ternary complexe stability through the measurement of kinetics associated with their formation and dissociation 193. Researchers have also constructed an extensive modeling framework that enables: (1) the assessment of PROTACs based on precise degradation metrics, (2) the guidance of crucial compound parameters, and (3) the establishment of a connection between degradation and downstream pharmacodynamic effects 194. Zhao *et al*. introduced a simple kinetic model that aligns well with the kinetics of PROTAC-induced protein degradation and the concentration-dependent degradation rate 195. These findings, strongly confirmed by experimental results, offer valuable insights into the design of PROTACs, aiming for enhanced selectivity and efficacy. Moreover, a more powerful and effective strategy entails the rational design of potent PROTAC molecules by leveraging the structural of POI-PROTAC-E3 ternary complex. Additionally, it is worth noting that the available repertoire of exploitable E3 ligases is currently quite restricted, and expanding this pool could potentially yield PROTACs with selectivity profiles. Finally, the emergence of resistance mechanisms in cancer cells, as reported in recent studies, presents imminent challenges and introduces new dimensions that necessitate careful consideration within the realm of PROTAC research 189.

In recent years, medicinal chemists have a preference for exploring an enhanced class of degraders that surpass the classical PROTACs, bypassing the inherent shortcomings associated with PROTACs and improve the efficacy targeted protein degradation 196. Therefore, a variety of other strategies for TPD have also been documented, which serve as complementary methods to PROTACs and enable complete protein degradation via both proteasomal and lysosomal pathways—such as molecular glues, SNIPERs, HyT, dTAG, Trim-Away, and other lysosome-based strategies.

In this review, we aim to elucidate recent progress in the application of protein degradation technology, specifically PROTACs, in the realm of epigenetic drug exploration. We have also briefly discussed the mechanisms behind different types of protein degraders. For most of the degraded epigenetic proteins, mainly 'readers', the advancement of PROTACs targeting less investigated epigenetic proteins and employing them as chemical tools can contribute to a investigate their function in diseases. Furthermore, enhancements in these chemical tools, which were initially established through the groundbreaking investigations outlined in this review, are also anticipated.

## Figures and Tables

**Table 1:** Representative epigenetic targets susceptible to degradation.

Epigenetic targets	TPD technologies	Ref
BRD4	PROTAC	23-41
	Ab-PROTAC	42-44
	CLIPTAC	15
	ATTEC	45
	pc-PROTAC	46, 47
	SNIPER	48
BRD2	photoPROTAC	49
	Trivalent PROTAC	50
BRD9	PROTAC	51-53
BRD2/3/4	PROTAC	54-56
CBP/p300	PROTAC	57
SMARCA	PROTAC	58-60
HDAC6	PROTAC	61-67
HDAC8	PROTAC	68-72
SIRT2	PROTAC	73-75
EED	PROTAC	76-78
EZH2	Hyt	79, 80
	PROTAC	81-84
WDR5	PROTAC	85-87
NSD2	PROTAC	88
NSD3	PROTAC	89, 90
EGFR	PROTAC	91-96
	ha-PROTAC	97, 98
	LYTAC	99
USP7	PROTAC	100, 101

## Abbreviations

ABPP: activity-based protein profiling
Ab-PROTACs: antibody-PROTACs
ADCs: antibody drug conjugates
AF9: ALL1-fused gene from chromosome 9
ALK: anaplastic lymphoma kinase
AML: acute myeloid leukemia
AR: androgen receptor
AREG: amphiregulin
ASGPR: asialoglycoprotein receptor
ATPases: ATP-dependent helicases
ATTECs: autophagosome-tethering compounds
AUTACs: autophagy-targeting chimeras
BAF: BRG1/BRM-associated factor
BET: bromodomain and extra terminal
BL: Burkitt's lymphoma
BMI1: B-cell specific Moloney murine leukemia virus integration site 1
BRD2: bromodomain-containing protein 2
BRD3: bromodomain-containing protein 3
BRD4: bromodomain-containing protein 4
BRD7): bromodomain-containing protein 7
BRDs: bromodomains
BRDT: bromodomain motif-containing protein
BTK: Bruton's tyrosine kinase
CBP: CREB-binding protein
CDDO-Me: bardoxolone methyl
cIAP1: cellular inhibitors of apoptosis protein 1
CI-M6PR: cation-independent mannose-6-phosphate receptor
CLIPTACs: click-formed proteolysis targeting chimeras
CRBN: cereblon
CRPC): castration-resistant prostate cancer
c-Myc: cellular Myc
DB: diffuse large B-cell lymphoma
DCs: dendritic cells
DMNB: 4,5-dimethoxy-2-nitrobenzyl
DNMTis: DNA methyltransferase inhibitors
DNMTs: DNA methyltransferases
dTAG: degradation TAG
DUB: deubiquitinating enzyme
EED: embryonic ectoderm development
EGFR: epidermal growth factor receptor
ENL: eleven-nineteen-leukemia
ER: estrogen receptor
EZH1/2: zeste homolog 1/2
FDA: Food and Drug Administration
FOLR1: folate receptor alpha
GCN5: general control nonderepressible 5
H2AK119ub: mono-ubiquitination of histone H2A at Lys119
HALG: hypoxia-activated leaving group
HATs: histone acetyltransferases
HAUSP: herpes virus-associated ubiquitin-specific protease
ha-PROTACs: hypoxia-activated PROTACs
HCC: hepatocellular carcinoma
HDACis: histone deacetylase inhibitors
HDACs: histone deacetylases
HIF-1α: hypoxia-inducible factor-1α
HMTs: histone methyltransferases
KDMs: histone demethylases
HNSCC: head and neck squamous cell carcinoma
HyT: hydrophobic tagging
MCL): mantle cell lymphoma
IAP: inhibitor of apoptosis protein
IDO1: indoleamine-2,3-dioxygenase-1
IKZF: Ikaros zinc finger
IMiDs: immunomodulatory drugs
IP: intraperitoneal
KATs: lysine acetyltransferases
KDM5C: lysine demethylase 5C
KEAP1: Kelch-like ECH-associated protein 1
KO: knockout
LPS: lipopolysaccharide
LTRs: lysosome-targeting receptors
mAbs: monoclonal antibodies
MEK: mitogen-activated protein kinase
MG: molecular glue
MLL: mixed lineage leukemia
MM: multiple myeloma
MOA: mechanism of action
MW: molecular weight
MYST: M oz, Y bf2/Sas3, S as2, and T ip60
NAD: nicotinamide adenine dinucleotide
NSG): NOD-scid IL2rγnull
NPOM: nitropiperonyloxymethyl
NSCLC: non-small cell lung cancer
NTR: nitroreductase
PBAF: polybromo-associated BAF
PCAF: P300/CBP-associated factor
PcG: polycomb group
PCGF1-6: PcG RING fingers 1-6
pc-PROTACs: photo-caged PROTACs
PDAC: pancreatic ductal adenocarcinoma
PDX: patient-derived xenograft
PD-L1: programmed death receptor ligand 1
PEG: polyethylene glycol
PG: phenyl glutarimide
PHOTACs: PHOtochemically TArgeting Chimeras
photoPROTAC: photo-switchable PROTAC
PIM1: pro-viral integration site of the Moloney murine leukemia virus 1
PK: pharmacokinetics
polo-like kinase 1): PLK1
POI: protein of interest
PPIs: protein-protein interactions
PRC1: polycomb repressive complex 1
PRC2: polycomb repressive complex 2
PRMT5: protein arginine methyltransferase 5
PRMTs: protein arginine methyltransferases
PROTACs: proteolysis-targeting chimeras
pro-PROTACs: prodrug-based PROTACs
PSS: photostationary state
Rb: retinoblastoma
RING1A/B: really interesting new gene 1A/B
RNAi: RNA interference
RNF4: ring finger protein 4
RNF114: ring finger protein 114
RT-PROTACs: radiotherapy-triggered PROTACs
SET: Su (var)3-9, Enhancer-of-zeste and Trithorax
SMARCA: SWI/SNF related, matrix associated, actin-dependent regulator of chromatin, subfamily A
SMIs: small-molecule inhibitors
SNIPER: specific and nongenetic IAP‐dependent protein eraser
STEAP1: six transmembrane epithelial antigen of the prostate 1
SUZ12: suppressor of the zeste 12 protein homolog
SWI/SNF: switch/sucrose non-fermentable
Taf14: transcription initiation factor TFIID subunit 14
TCO: *trans*-cyclooctene
TIF1α: transcriptional intermediary factor 1α
TKIs: tyrosine kinase inhibitors
TNBC: triple-negative breast cancer
TPD: targeted protein degradation
TPSA: topological polar surface area
TRIM24: tripartite motif-containing 24
TSCC: tongue squamous cell carcinoma
Tz-Thalidomide: tetrazine-tagged E3 ligase recruiter
T-ALL: T-cell acute lymphoblastic leukemia
Ub: ubiquitin
UPS: ubiquitin-proteasome system
VHL: Von Hippel-Lindau
MDM2: mouse double minute 2
WDR5: WD40 repeat domain protein 5
WHIM: Washington University Human-in-Mouse
USP7: ubiquitin-specific protease 7
UV: ultraviolet
XIAP: X-linked inhibitor of apoptosis protein
